# Iterative Synthesis of Pyrene–Coronene Molecular Graphene Nanoribbons

**DOI:** 10.1002/anie.6619000

**Published:** 2026-06-23

**Authors:** Miguel A. Medel, Guanzhao Wen, Adrián Morón‐Blanco, Mischa Bonn, Hai I. Wang, Manuel Melle‐Franco, Aurelio Mateo‐Alonso

**Affiliations:** ^1^ POLYMAT, Department of Applied Chemistry University of Basque Country (EHU) Donostia‐San Sebastián Spain; ^2^ Max Planck Institute For Polymer Research Mainz Germany; ^3^ Nanophotonics, Debye Institute For Nanomaterials Science Utrecht University Utrecht the Netherlands; ^4^ CICECO—Aveiro Institute of Materials, Department of Chemistry University of Aveiro Aveiro Portugal; ^5^ Ikerbasque, Basque Foundation For Science Bilbao Spain

**Keywords:** graphene nanoribbons, nanographenes, polycyclic aromatic hydrocarbons

## Abstract

The synthesis of molecular, or monodisperse, graphene nanoribbons with full atomic precision is essential for establishing fundamental structure–property relationships, validating theoretical predictions, and meeting the property requirements for the diverse potential applications of graphene nanoribbons. The synthesis of undoped molecular graphene nanoribbons remains challenging, as many edge topologies have yet to be realized and the reported structures still exhibit limited lengths. This is mostly because iterative synthetic methods for undoped molecular GNRs remain practically undeveloped, with current approaches relying predominantly on noniterative strategies. The iterative synthesis of a new family of undoped molecular GNRs, featuring both a novel edge topology and an unprecedented length, is reported. These nanoribbons are accessed through a borylation/Suzuki/cyclodehydrogenation reaction sequence, in which the Suzuki and cyclodehydrogenation are merged into one transformation, giving rise to an effective two‐step iteration sequence. The resulting pyrene–coronene graphene nanoribbons exhibit strong absorption and high fluorescence efficiency, with molar absorptivity and fluorescence brightness values on the order of 10^5^ M^−1^ cm^−1^, and an intrinsic charger carrier mobility of 475 ± 32 cm^2^ V^−1^ s^−1^.

## Graphical abstract

An iterative approach delivers a new family of undoped, cove‐edged graphene nanoribbons with lengths up to 9.1 nm that exhibit both molar absorptivity and fluorescence brightness values on the order of 10^5^ M^−1^ cm^−1^ and an intrinsic charge‐carrier mobility of 475 ± 32 cm^2^ V^−1^ s^−1^.

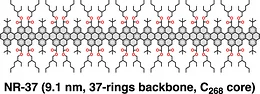

## 1 Introduction

Graphene nanoribbons (GNRs) are quasi‐one‐dimensional cutouts of graphene that have shown great promise as materials for a broad range of electronic, photonic, and spintronic applications [1, 2, 3, 4]. The properties of GNRs are governed by quantum confinement and edge effects, making parameters such as edge topology, width, length, and heteroatom doping powerful levers to precisely tailor their electronic structure. The synthesis and exploration of diverse edge topologies is an essential aspect of GNR research, as the edge structure governs key electronic and magnetic properties, including metallicity, bandgap, and spin‐polarisation [1]. By accessing armchair, zigzag, cove, chevron, or chiral edges, it becomes possible to engineer bandgaps, control spin states, and introduce topologically protected edge states relevant for quantum and spintronic applications. Some edges (e.g., cove and fjord) can also distort the otherwise planar GNR backbone, which can increase solubility and, in some cases, give rise to chiroptical properties [5, 6, 7, 8, 9, 10]. While parameters such as length, width, and heteroatom doping strongly influence the energy levels and edge states of GNRs [1, 11, 12, 13, 14], length influences absorptivity [15, 16, 17, 18], charge transport [18, 19], singlet fission [20], gel microstructure [21], and enables device integration [22, 23, 24], among others [25, 26]. Consequently, the development of synthetic methods that deliver GNRs with atomic precision across all these structural variables is essential for establishing their fundamental structure–property relationships, validating theoretical predictions, and meeting the property requirements of different potential applications.

**FIGURE 1:**
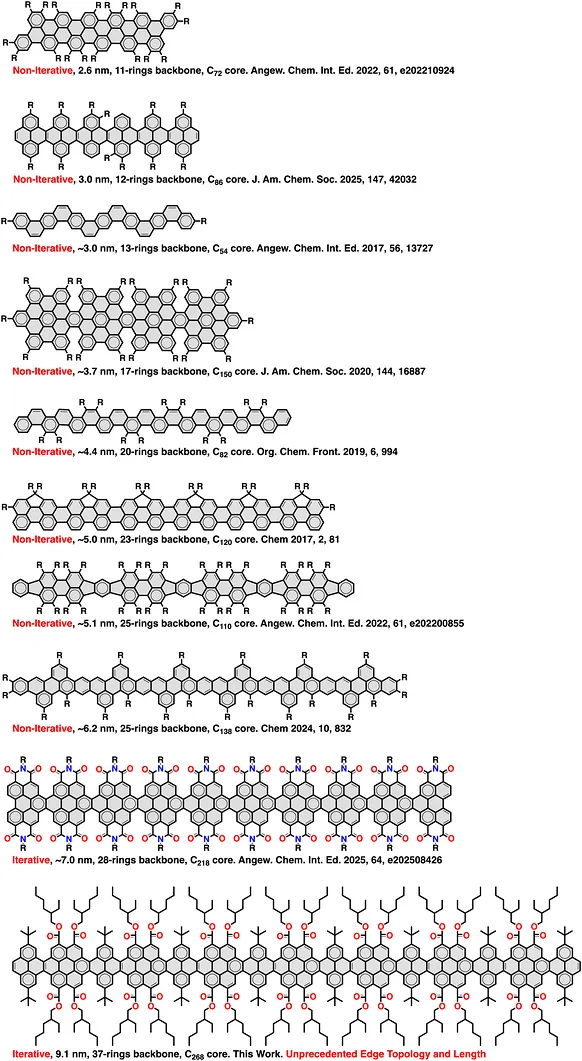
Representative families of undoped molecular GNRs featuring different edge topologies, highlighting the longest member of each family together with **NR‐37**, reported in this work.

Advances in organic chemistry have enabled the synthesis of molecular or monodisperse GNRs with full atomic precision over all the above‐mentioned structural parameters. An advantage of solution‐phase organic synthesis is that it provides series of monodisperse GNRs in quantities ranging from tenths to hundreds of milligrams, which are sufficient to carry out a systematic investigation of their fundamental properties using a wide variety of spectroscopic and redox characterization techniques. Such organic synthetic methods have already delivered a broad diversity of GNRs with different edge topologies (Figure 1) [7, 8, 10, 15, 16, 17, 18, 19, 27, 28, 29, 30, 31, 32, 33, 34, 35, 36, 37, 38, 39, 40, 41, 42, 43, 44, 45, 46, 47].

Despite the impressive progress in the synthesis of molecular GNRs, many edge topologies have not yet been realized. Moreover, the undoped molecular GNRs reported to date [7, 10, 27, 31, 34, 36, 42, 43, 45, 46, 47] are substantially shorter than their nitrogen‐doped counterparts [15, 16, 17, 18, 19, 32, 37]. This is mostly because iterative synthetic methods for undoped molecular GNRs remain practically undeveloped [10], with current approaches relying predominantly on noniterative strategies, including both modular and nonmodular multistep routes (Figure 1).

Herein, we describe the iterative synthesis of a new family of undoped molecular GNRs constituted of alternating fused pyrene and coronene units, featuring both a novel edge topology and a record length (Figure 1). To access this family of molecular GNRs, we have developed an iterative synthetic methodology based on an unprecedented borylation/Suzuki/cyclodehydrogenation sequence, in which the Suzuki coupling and the cyclodehydrogenation are merged into a single cascade reaction, effectively transforming a three‐step iterative process into a two‐step one. The implementation of this two‐step iterative workflow has afforded a series of undoped molecular GNRs, the longest of which (**NR‐37**) features a 9.1 nm backbone consisting of 37 linearly fused rings (C_268_ core), representing the longest undoped molecular GNR reported to date (Figure 1) and exceeding the size of previously known giant undoped nanographenes (core >200 atoms) such as C_216_ [48], C_222_ [49, 50, 51], and C_258_ [52]. Because of the cove regions at the pyrene–coronene junctions and the proximity of the tetraester functionalities, this type of molecular pyrene–coronene GNR adopts a stable helical conformation which, together with the selected solubilizing groups, ensures sufficient solubility to enable purification by conventional flash chromatography as well as detailed structural, optoelectronic, and redox characterization. This family of molecular GNRs exhibit strong absorption and high fluorescence efficiency, with molar absorptivity and molar fluorescence brightness values on the order of 10^5^ M^−1^ cm^−1^, and an intrinsic charger carrier mobility of 475 ± 32 cm^2^ V^−1^ s^−1^, which ranks among the highest values for undoped GNRs.

## 2 Results and Discussion

### 2.1 Synthesis

**SCHEME 1:**
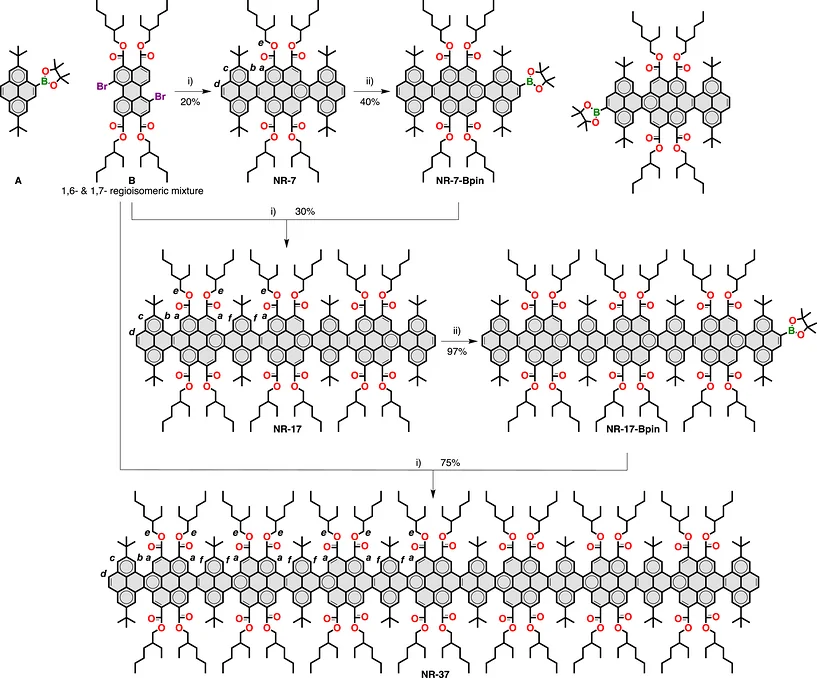
Synthesis of **NR‐7**, **NR‐17**, and **NR‐37**. (i) Pd(PPh_3_)_4_, K_2_CO_3_, THF, water, 80°C, 1 day; (ii) [Ir(OMe)COD]_2_, B_2_pin_2_, Me_4_phen, cyclohexane, reflux, 1 day. The lettering indicates the proton assignments on the ^1^H NMR spectra in Figure 2.

The synthesis of the pyrene–coronene molecular GNRs starts from 2,7‐di‐*tert*‐butyl‐4‐pinacolboronate‐pyrene **A** and tetrakis(2‐ethylhexyl)‐1,X‐dibromoperylene‐3,4,9,10‐tetracarboxylate **B** (mixture of isomers 1,6 and 1,7) (Scheme 1), which were obtained by iridium‐catalyzed borylation of pyrene [6] and by bromination of perylenebisimide [18], respectively, following methods described in the literature. The reaction between **A** and **B** using Pd(PPh_3_)_4_, K_2_CO_3_ in a THF/water mixture yielded directly **NR‐7** (20%) after 1 day (Scheme 1), in addition to the double Suzuki coupling product (50%). This unexpected direct formation of **NR‐7** formally corresponds to a Suzuki/cyclodehydrogenation reaction, although it proceeds through a cascade or domino process [53] rather than through two consecutive Suzuki and cyclodehydrogenation steps. This was demonstrated by a control experiment in which the corresponding double Suzuki coupling product was subjected to identical reaction conditions (Figure S1). Under these conditions, the double Suzuki coupling product remained essentially unreacted, and only trace amounts of **NR‐7** were detected in the crude mixture. This result indicates that cyclodehydrogenation takes place preferentially through an intermediate generated during the coupling step and hence that the transformation is the result of a cascade process. One plausible scenario is that an organopalladium intermediate generated during the Suzuki coupling step remains sufficiently reactive to progress to the cyclodehydrogenated product through a cascade pathway, similarly to the annulative dimerization mechanism proposed for phenanthrene derivatives [54]. This is in contrast with a previous report of a related transformation [55], where the cyclodehydrogenated product can be obtained directly from the isolated cross‐coupling product. **NR‐7** was then borylated (40%) using bis(pinacolato)diboron (B_2_pin_2_) and cyclooctadiene iridium methoxide dimer in the presence of 3,4,7,8‐tetramethyl‐1,10‐phenanthroline (Me_4_phen) ligand. The resulting **NR‐7‐Bpin** was allowed to react with compound **B** under the same Pd‐catalyzed conditions for **NR‐7**, yielding **NR‐17** (30%) after the same formal Suzuki/cyclodehydrogenation cascade reaction (Scheme 1). At this stage, **NR‐17** was borylated using the same reaction conditions for **NR‐7**, yielding **NR‐17‐Bpin** in a much better yield (97%). Finally, the reaction between **NR‐17‐Bpin** and **B** yielded **NR‐37** (75%) after the formal Suzuki/cyclodehydrogenation cascade reaction (Scheme 1).

**FIGURE 2:**
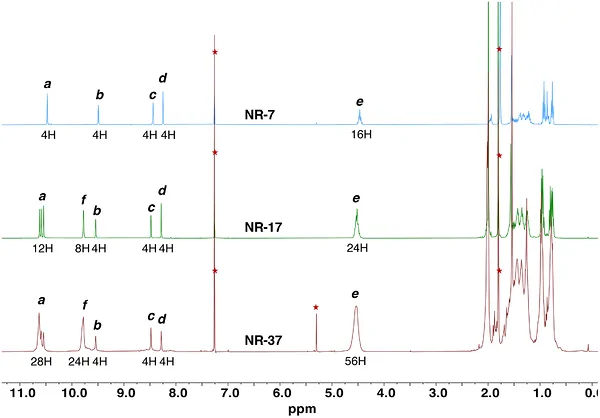
^1^H NMR spectra of **NR‐7**, **NR‐17,** and **NR‐37** in CDCl_3_ at room temperature. Stars indicate signals from residual solvents.

All GNRs are soluble in common organic solvents, such as chloroform, dichloromethane, toluene, and 1,2‐dichlorobenzene, so we were able to determine their structure by ^1^H and ^13^C NMR spectroscopy in CDCl_3_ at room temperature, and by mass spectrometry. The NMR signals in the aromatic and aliphatic region are consistent with the structure. For instance, integration of the ^1^H NMR spectra can be used to unambiguously determine the length of the GNR. For example, signals *b*, *c*, and *d* that correspond to the terminal pyrene segments (the assignments in Figure 2 correspond to the lettering shown in Scheme 1) show a constant integration of 4, 4, and 4 protons, respectively, in all GNRs. In contrast, the signal *e* corresponding to the methylenes directly attached to the perylene tetraester segments integrates 16, 24, and 56 protons, respectively, for **NR‐7**, **NR‐17** and **NR‐37** (Figure 2). InThis trend can also be observed in the integration of other signals (*a* and *f*) that also increase proportionally to the number of protons with respect to the signals of the terminal pyrene segments (*b*, *c* and *d*) as the length increases. Matrix‐assisted laser desorption/ionization time‐of‐flight mass spectrometry (MALDI‐TOF MS) shows the expected molecular‐ion peak distributions for the GNRs.

### 2.2 Structure

**FIGURE 3:**
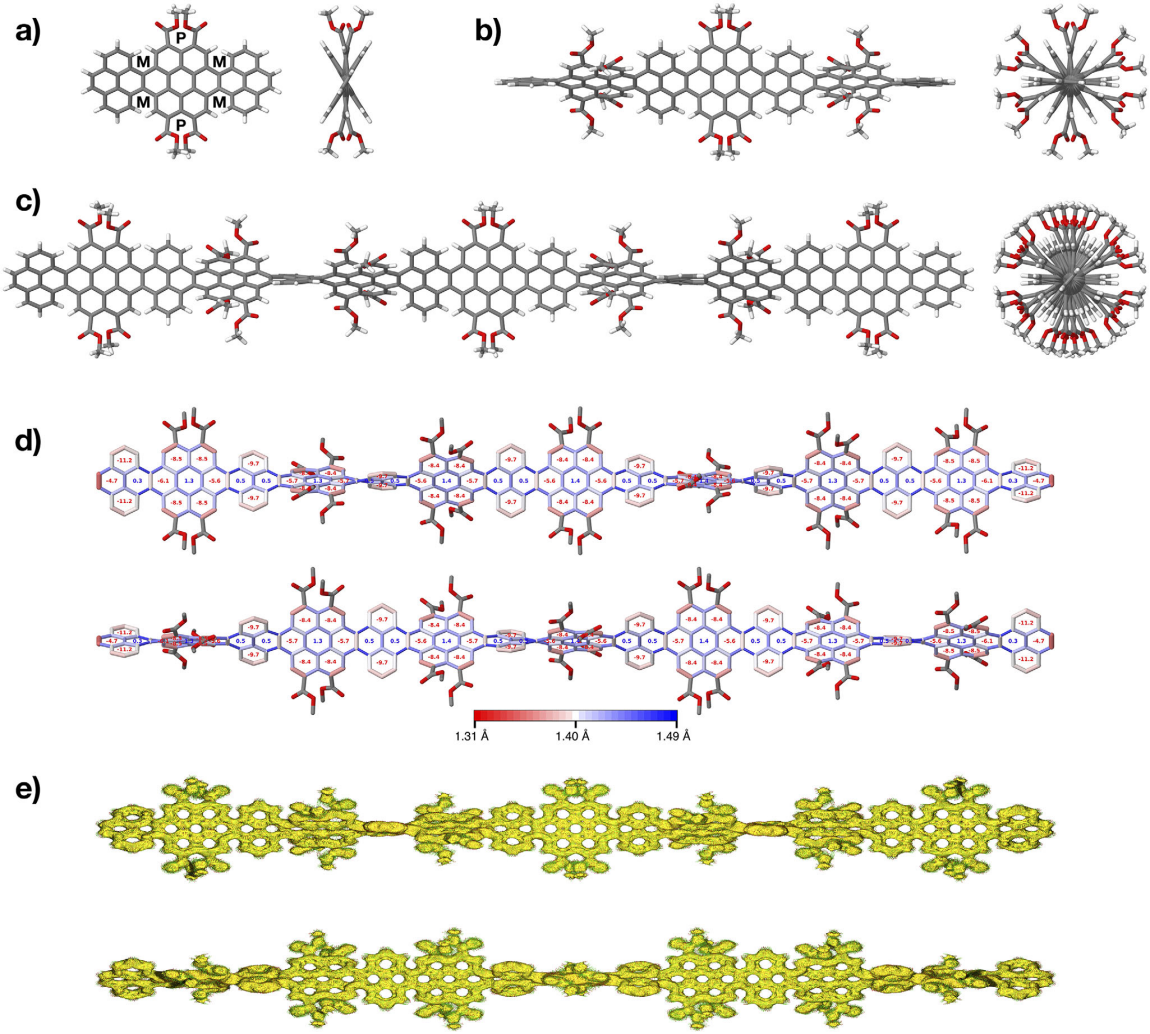
Longitudinal and axial views of the computed M06‐2X‐6‐31G(d,p) structure of (a) **NR‐7‐H**, (b) **NR‐17‐H**, and (c) **NR‐37‐H**. (d) Bond length analysis, NICS(0) and (e) ACID plots with two alternative orientations for **NR‐37‐H**.

Theoretical calculations illustrate the twisted conformation of this type of pyrene–coronene GNRs (Figure 3a–c). The calculations were carried out on simplified models, namely **NR‐7‐H**, **NR‐17‐H** and **NR‐37‐H**, where the 2‐ethylhexyl ester and *tert*‐butyl groups present in **NR‐7**, **NR‐17** and **NR‐37** were replaced by methyl ester groups and hydrogen atoms, respectively, for computational efficiency. The conformational landscape of **NR‐7‐H**, which was selected as a model for the alternating pyrene–coronene segments present in all the GNRs, was investigated with a global conformation search methodology based on metadynamics with the CREST software with tight‐binding GFN Hamiltonians. These calculations show two twists at the cove regions located at the pyrene–coronene junctions, because of the steric congestion generated by the inner hydrogen atoms (Figure 3a). Furthermore, there is an additional twist in between the tetraester groups because of their proximity (Figure 2C). **NR‐7‐H** preferentially adopts two enantiomeric helical conformations (designated as *P*,*M*,*P* and *M*,*P*,*M*, Figure S2) that interconvert rapidly at room temperature because of the low interconversion barriers typical of these systems [6, 18, 56, 57, 58]. These *P*,*M*,*P* and *M*,*P*,*M* helical conformations remain unaltered regardless of the multiple rotamers arising from the tetraester moieties. The alternated conformations (*P*,*P*,*M* and *M,M,P*) are not thermodynamically accessible illustrating that the helical arrangement is the most stable. This helical pattern extends over the longer **NR‐17‐H** and **NR‐37‐H** (Figure 3b,c), which yield estimated end‐to‐end lengths of 1.7, 4.2 and 9.1 nm for **NR‐7‐H**, **NR‐17‐H,** and **NR‐37‐H**, respectively.

The dominant resonance structures of all GNRs are best described by Clar's sextet rule with a biphenyl group (two sextets) on the pyrene segments and a coronene group (three sextets) on the coronene segments, as shown in Scheme 1. This assignment is supported by bond‐length alternation analysis (Figures 3d and S3), which reveals nearly aromatic distances in the off‐linear pyrene rings and in the coronene residues, double‐bond distances in the K‐region of the terminal pyrenes, and single‐bond distances in the pyrene rings adjacent to the coronenes. Consistently, aromatic negative NICS(0) values (shown in red in Figures 3d and S3) are found for the off‐linear pyrene rings and the outer rim coronene rings, whereas nonaromatic positive, but close to zero, values (shown in blue in Figures 3d and S3) are observed for the pyrene rings adjacent to the coronenes and for the central coronene rings. These trends are further corroborated by NICS(1) calculations (Figure S4). Moreover, the anisotropy of the induced current density (ACID) plots of **NR‐7‐H**, **NR‐17‐H**, and **NR‐37‐H** (Figures 3e and S5) display a diamagnetic ring current around the ribbon periphery, as previously described for other types of molecular GNRs [16, 17].

### 2.3 Optoelectronic and Redox Properties

**NR‐7**, **NR‐17**, and **NR‐37** give rise to pale red solutions in dichloromethane. Their absorption spectra show similar features across all GNRs: a broad band at ∼400 nm and a second band with clear vibronic features at ∼530 nm, which we refer to as the *β* and *ρ* bands, respectively. The absorption spectra of **NR‐17** and **NR‐37** are practically superimposable and exhibit a 6–10 nm redshift relative to **NR‐7**, as illustrated by the maxima of the *β* (391, 400, and 401 nm for **NR‐7**, **NR‐17**, and **NR‐37**, respectively) and *ρ* (530, 536, and 536 nm, respectively) bands. The minimal spectral evolution beyond **NR‐17** indicates that the electronic structure has essentially reached the quasi‐infinite limit, suggesting that quantum confinement is particularly effective for this combination of edge topology and width. This behavior aligns with Clar's rule, where the stabilization arising from the localized π‐sextet distribution becomes dominant in longer ribbons. Despite this convergence in bandgap, the molar absorptivity (*ε*) increases with GNR length to reach a maximum of 586,000 M^−1^ cm^−1^ for **NR‐37** at the *β* band (*ε^β^* = 144,000, 380,000, 586,000 M^−1^ cm^−1^ and *ε^ρ^* = 31,300, 194,000, 310,000 M^−1^ cm^−1^ for **NR‐7**, **NR‐17**, and **NR‐37**, respectively).

Upon UV light excitation, solutions of **NR‐7**, **NR‐17**, and **NR‐37** in dichloromethane show intense yellow‐green fluorescence. The emission spectra of **NR‐17** and **NR‐37** are almost superimposable and exhibit a slight 4 nm redshift relative to **NR‐7**. Notably, **NR‐37** exhibits a remarkably high fluorescence quantum yield (*Φ* = 51%) considering its length (*Φ* = 62%, 40%, and 51% for **NR‐7**, **NR‐17**, and **NR‐37**, respectively). Owing to the high *ε* and *Φ* values, the molar fluorescence brightness (*εΦ*) is remarkably high, on the order of 10^5^ M^−1^ cm^−1^, and increases with ribbon length (8.9 × 10^4^, 1.5 × 10^5^, and 3.0 × 10^5^ M^−1^ cm^−1^ for **NR‐7**, **NR‐17**, and **NR‐37**, respectively). These values are comparable to those of the state‐of‐the‐art inorganic quantum dots [59, 60].

The redox properties of **NR‐7**, **NR‐17**, and **NR‐37** were investigated by cyclic voltammetry in dichloromethane, using *n*Bu_4_NPF_6_ as supporting electrolyte and the ferrocene/ferrocenium couple as an internal reference (Fc/Fc^+^). All GNRs exhibit two reversible reduction waves (*E*_1/2_^I^ = –1.81, –1.82, and –1.82 V; *E*_1/2_^II^ = –2.04, –2.07, and –2.08 V for **NR‐7**, **NR‐17**, and **NR‐37**, respectively) and a reversible oxidation wave (*E*_1/2_^I’^ = 0.76, 0.79, and 0.78 V, for **NR‐7**, **NR‐17**, and **NR‐37**, respectively). In addition, oxidation waves can be seen for **NR‐17**, and **NR‐37** at the edge of the solvent‐supported electrolyte window.

### 2.4 Energy Levels and Orbitals

**FIGURE 4:**
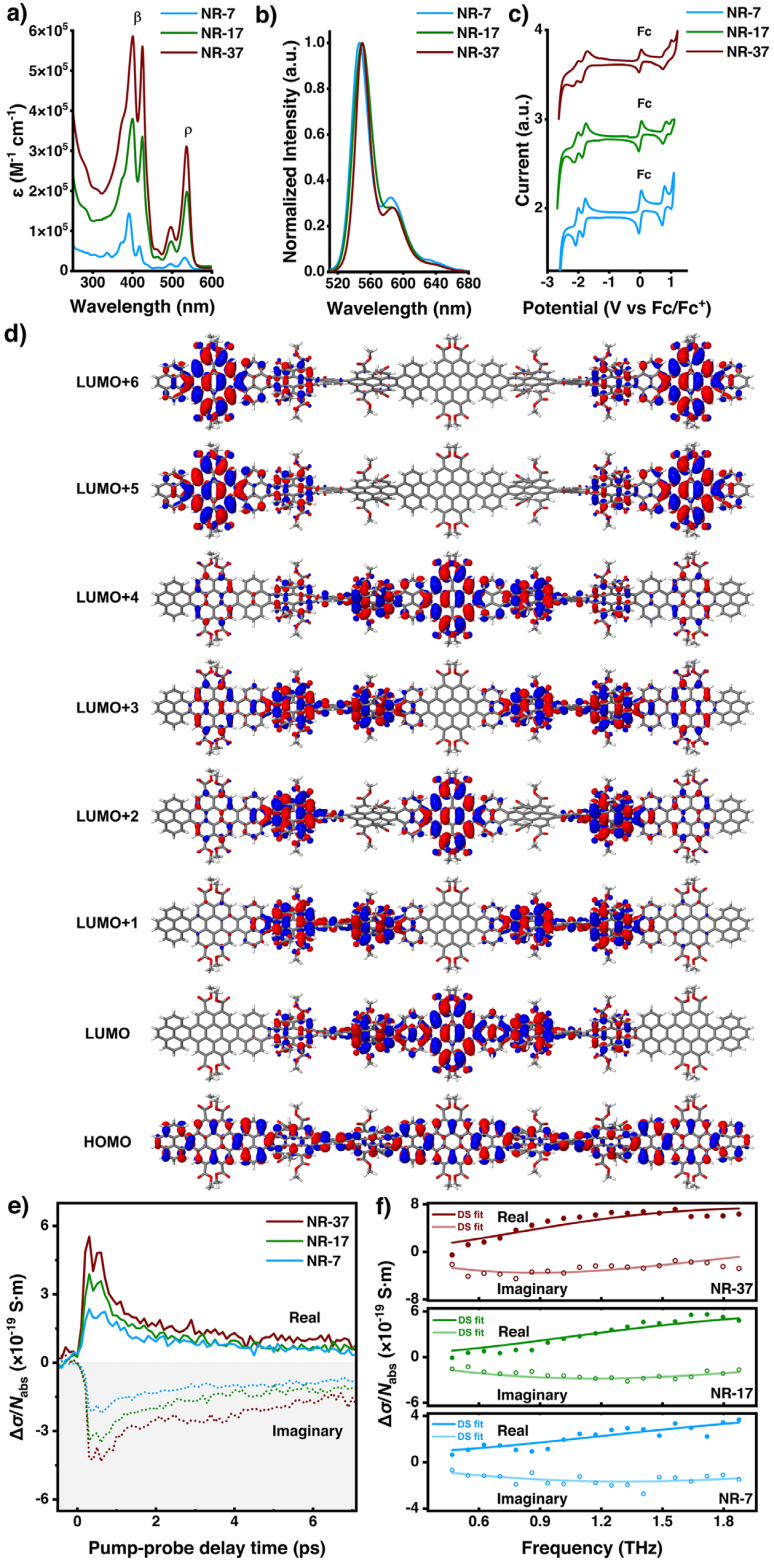
(a) UV‐vis absorption and (b) fluorescence spectra of the GNRs in CH_2_Cl_2_. (c) Cyclic voltammograms of the GNRs in *n*‐Bu_2_NPF_6_/CH_2_Cl_2_ (0.1 M). Potentials versus the ferrocene/ferrocenium couple (Fc/Fc^+^). (d) HOMO and quasidegenerate LUMOs of **NR‐37** plot with a 1 × 10^−2^ e Å^−3^ isovalue. (e) Comparison of time‐resolved THz photoconductivity normalized to the absorbed photon density (Δ*σ*/*N*_abs_) of GNRs with three different lengths following optical injection of charge carriers by excitation at 3.1 eV. (f) Frequency‐resolved complex THz photoconductivity of GNRs at 1.0 ps after the maximum THz photoconductivity. The solid line represents the Drude‐Smith (DS) model fits.

We inferred optical HOMO–LUMO gaps of 2.24, 2.20, and 2.20 eV from the onset of the lowest‐energy absorption, and electrochemical HOMO–LUMO gaps of 2.64, 2.61, and 2.60 eV by the potential difference between the onset of the first reduction and oxidation waves for **NR‐7**, **NR‐17**, and **NR‐37**, respectively. The obtained electrochemical LUMOs or electron affinities (∼ –3.0 eV) and electrochemical HOMOs or ionization potentials (∼ –5.6 eV) are nearly identical in all GNRs. The experimental energy levels are consistent with computed values, which also predict nearly identical HOMO–LUMO gaps (∼ 2.2 eV), LUMOs (∼ –2.4 eV) and HOMOs (∼ –5.2 eV) (Figures S6 and S7; Tables S1 and S2). This shows that the electronic structure quickly converges, with the bandgap approaching its saturation value already in short ribbons. In all cases, the HOMO is largely delocalized along the GNR backbone, even for the longest **NR‐37** (Figures 4d and S8–S10). In contrast, the spatial distribution of the LUMO evolves systematically with increasing ribbon length (Figures 4d and S8–S10). In **NR‐7**, the LUMO extends over the whole ribbon (Figure S8). In **NR‐17**, the LUMO is nearly degenerate with the next two molecular orbitals and confined to the inner pyrene–coronene–pyrene segment by the terminal pyrene segments, which remain essentially unpopulated (Figure S9). In the longer **NR‐37**, the LUMO is nearly degenerate with the next six molecular orbitals. All these orbitals exhibit enhanced electron density on the coronene moieties, progressively shifting toward the axial edges of the NR with increasing energy (Figures 4d and S10).

To calculate the band structure of an infinite pyrene–coronene GNR, a 1D periodic model derived from **NR‐37‐H** was constructed and optimized using the QuantumATK simulation package [61]. The model consists of a periodic unit cell of 3.75 × 4 × 4 nm^3^, corresponding structurally to a repeating **NR‐15‐H** segment with an axial periodicity of 3.75 nm. Calculations employed the BLYP functional augmented with Grimme's D2 empirical dispersion correction, PseudoDojo norm‐conserving pseudopotentials [62] and medium numerical basis sets for all atoms. The periodic highest occupied crystal orbital (HOCO) and lowest unoccupied crystal orbital (LUCO) are fundamentally similar to the molecular ones (Figures S11 and S12), confirming that the electronic structure rapidly converges to the quasi‐infinite limit with ribbon length and again highlighting that quantum confinement is particularly effective for this combination of edge topology and width. This observation is consistent with the minimal spectral evolution observed in the UV‐vis absorption spectra and, in turn, with the HOMO‐LUMO gap approaching its saturation value already in short ribbons. The band structure and the effective masses were computed with the B3LYP Hamiltonian under the same conditions. From the curvature at the maximum of the valence band (HOCO) and the minimum of the conduction band (LUCO), we computed the corresponding effective masses of the hole (*m*_h_* = 0.33) and the electron (*m*_e_* = 1.40) that yield a light reduced effective mass for charge carriers (m*) of 0.27.

### 2.5 Charge Transport

The charge transport properties of the molecular GNRs were further investigated using optical pump‐THz probe spectroscopy. Readers can find more experimental details in the previous similar studies by the same tool [18, 63, 64]. This noncontact technique is particularly powerful for evaluating the short‐range, or equivalently high‐frequency carrier mobility within finite‐sized nanostructures [65, 66, 67, 68, 69]. Following the injection of charge carriers by pulsed photoexcitation at 3.1 eV (pulse duration of ∼50 fs), the time‐resolved THz photoconductivity (Δ*σ*) for thin films of **NR‐7**, **NR‐17**, and **NR‐37** displays a similar temporal profile. In all cases, both the real and imaginary parts of photoconductivity exhibit a fast onset limited by the instrument response function, consistent with instantaneous free‐carrier generation after excitation, followed by a rapid decay (Figure 4e). This fast‐decay kinetics is typical of molecular GNRs and has been attributed to efficient carrier localization, for example, due to the formation of bound excitons and/or trapping at defect sites [70, 71]. Notably, the peak amplitude of photoconductivity exhibits a pronounced dependence on the molecular ribbon length, increasing from **NR‐7** (∼1.7 nm) to **NR‐37** (∼9.1 nm), consistent with the length‐dependent enhancement previously observed in GNR systems [18, 63]. To quantify the microscopic charge carrier transport property, the frequency‐dependent complex photoconductivity at ∼1.0 ps after the maximum photoconductivity for molecular GNR thin films was recorded, as shown in Figure 4f. The data were modeled using the Drude‐Smith (DS) model (solid lines in Figure 4f), which phenomenologically describes spatially confined charge transport through a backscattering parameter (*c*) and the scattering time (*τ*_DS_) [72, 73]. The inferred *τ*_DS_ for **NR‐7**, **NR‐17**, and **NR‐37** is 50 ± 5, 55 ± 5, and 73 ± 5 fs, respectively, reflecting end‐limited backscattering effects, in line with previous work [18, 63]. This result suggests that for infinitely long ribbons, the charge‐carrier scattering time equals or exceeds 73 fs and be effectively free of backscattering. Based on these discussions and the effective mass (m*) derived from the DFT calculations, we determined the intrinsic charge carrier mobility (*μ*, *μ* = *eτ*_DS_/m*) value of 475 ± 32 cm^2^ V^−1^ s^−1^ for **NR‐37** that corresponds to the lower‐boundary of intrinsic charge carrier mobility for this family of pyrene–coronene molecular GNRs. This *μ* value is one order of magnitude higher than the values observed in conjugated polymers such as P3HT [74, 75] (*μ* ∼ 10^1^ cm^2^ V^−1^ s^−1^), lies on the same order of magnitude (10^2^ cm^2^ V^−1^ s^−1^) as the highest values reported for the state‐of‐the‐art polydisperse GNRs [66, 67, 76, 77, 78, 79, 80, 81] and porphyrin nanoribbons [82, 83], and is especially remarkable considering the narrow width of these GNRs [18, 67, 76, 77, 78].

## 3 Conclusion

In summary, we have developed a novel iterative synthetic strategy that enables the efficient synthesis of a new family of undoped molecular GNRs featuring an unprecedented edge topology and length. This was made possible by introducing an iterative borylation/Suzuki/cyclodehydrogenation workflow in which the Suzuki coupling and cyclodehydrogenation steps are merged into a single cascade reaction, thereby transforming a three‐step iterative process into a two‐step one. This family of GNRs exhibits both strong absorption and high fluorescence efficiency, with molar fluorescence brightness values (*εΦ*) on the order of 10^5^ M^−1^ cm^−1^, comparable to those of the state‐of‐the‐art inorganic quantum dots [59, 60]. In addition, it shows a high intrinsic charge‐carrier mobility of 475 ± 32 cm^2^ V^−1^ s^−1^, which ranks among the highest values reported for the state‐of‐the‐art polydisperse GNRs [66, 67, 76, 77, 78, 79, 80, 81] and porphyrin nanoribbons [82, 83]. Overall, this work establishes a synthetically efficient route to long, undoped, cove‐edged molecular GNRs that combine exceptional structural precision, optical brightness, and intrinsic charge‐transport performance. These results open new avenues for the design of tunable π‐conjugated nanostructures for next‐generation optoelectronic and quantum devices.

## Author Contributions

**Miguel A. Medel**: investigation, writing – review and editing. **Guanzhao Wen**: investigation, writing – review and editing. **Adrián Morón‐blanco**: investigation. **Mischa Bonn**: investigation, writing – review and editing. **Hai I. Wang**: investigation, writing – review and editing. **Manuel Melle‐franco**: investigation, writing – review and editing. **Aurelio Mateo‐Alonso**: investigation, conceptualization, writing – original draft.

## Conflicts of Interest

The author declare no conflicts of interest.

## Supporting information



The authors have cited additional references within the Supporting Information [84]. **Supporting File 1**: anie73272‐sup‐0001‐SuppMat.pdf.

## Data Availability

The data that supports the findings of this study are available in the supplementary material of this article.
