# Supplementary material for: Iterative Synthesis of Pyrene–Coronene Molecular Graphene Nanoribbons
Source: Angew Chem Int Ed Engl. 2026 Jun 23;65(35):e6619000. doi: 10.1002/anie.6619000 (PMC13502667; doi:10.1002/anie.6619000)
Supplement: Supplementary file 1 [file ANIE-65-e6619000-s001.pdf]

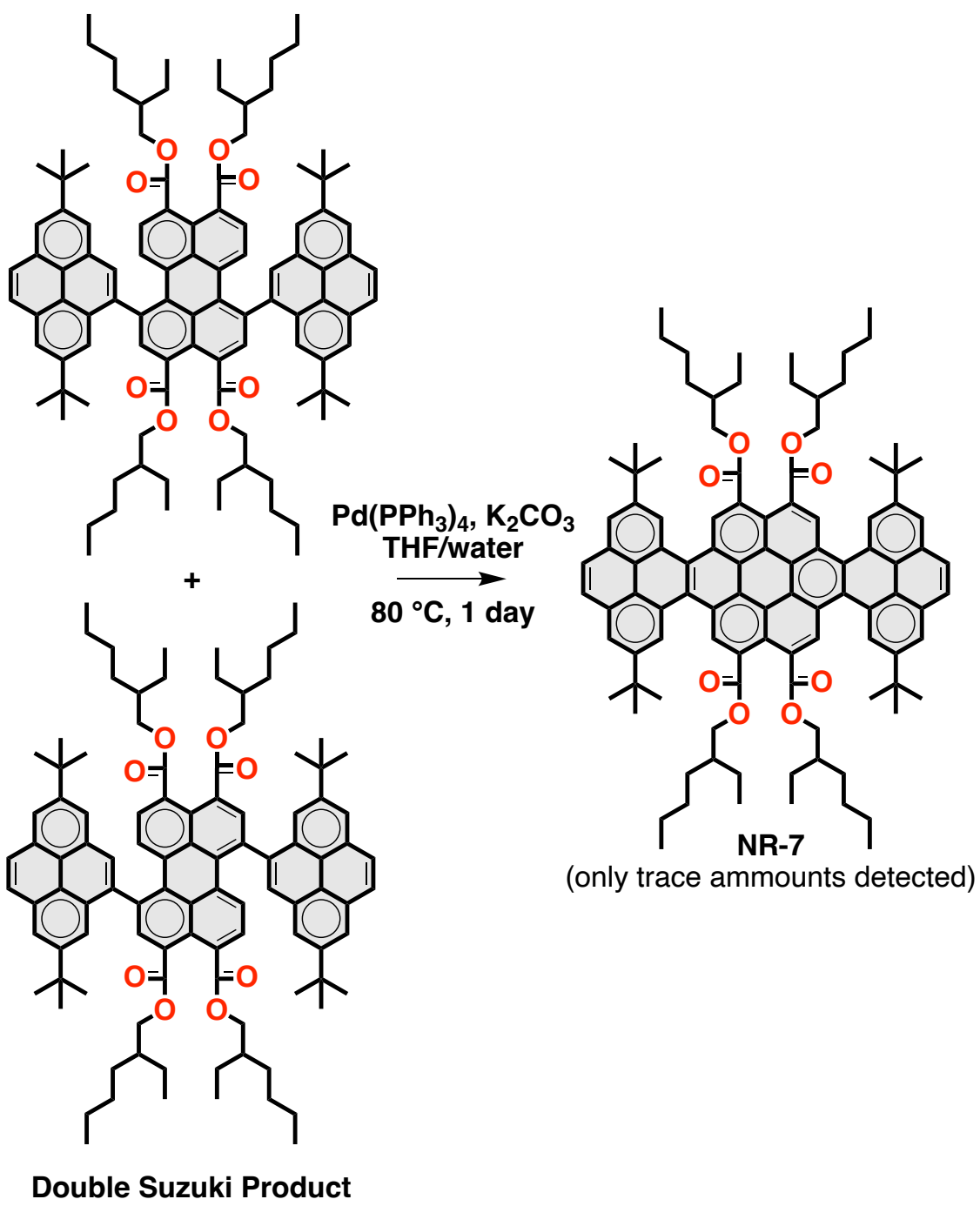

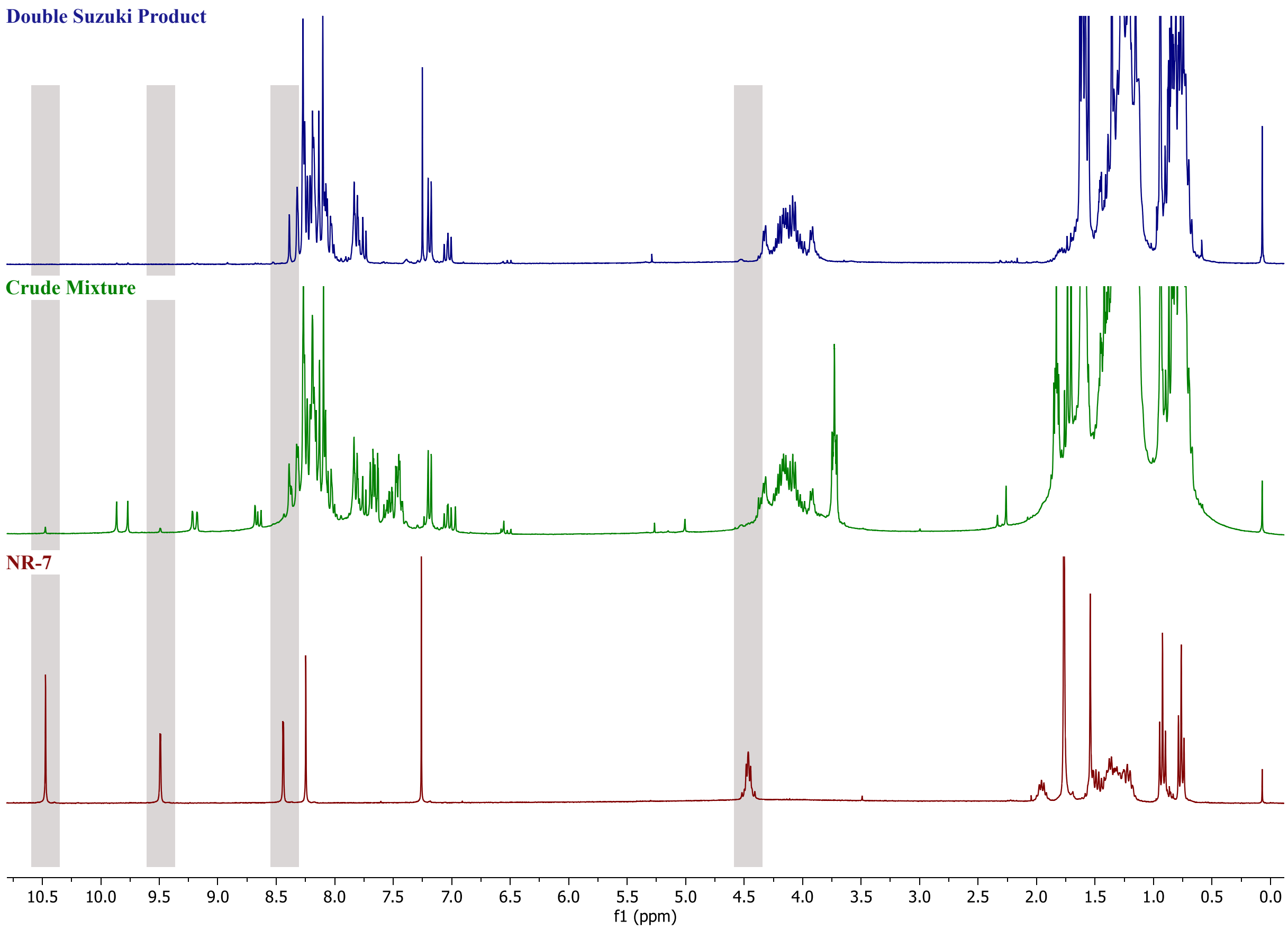

Figure S1. Control experiment starting from the corresponding double Suzuki coupling product using identical conditions.

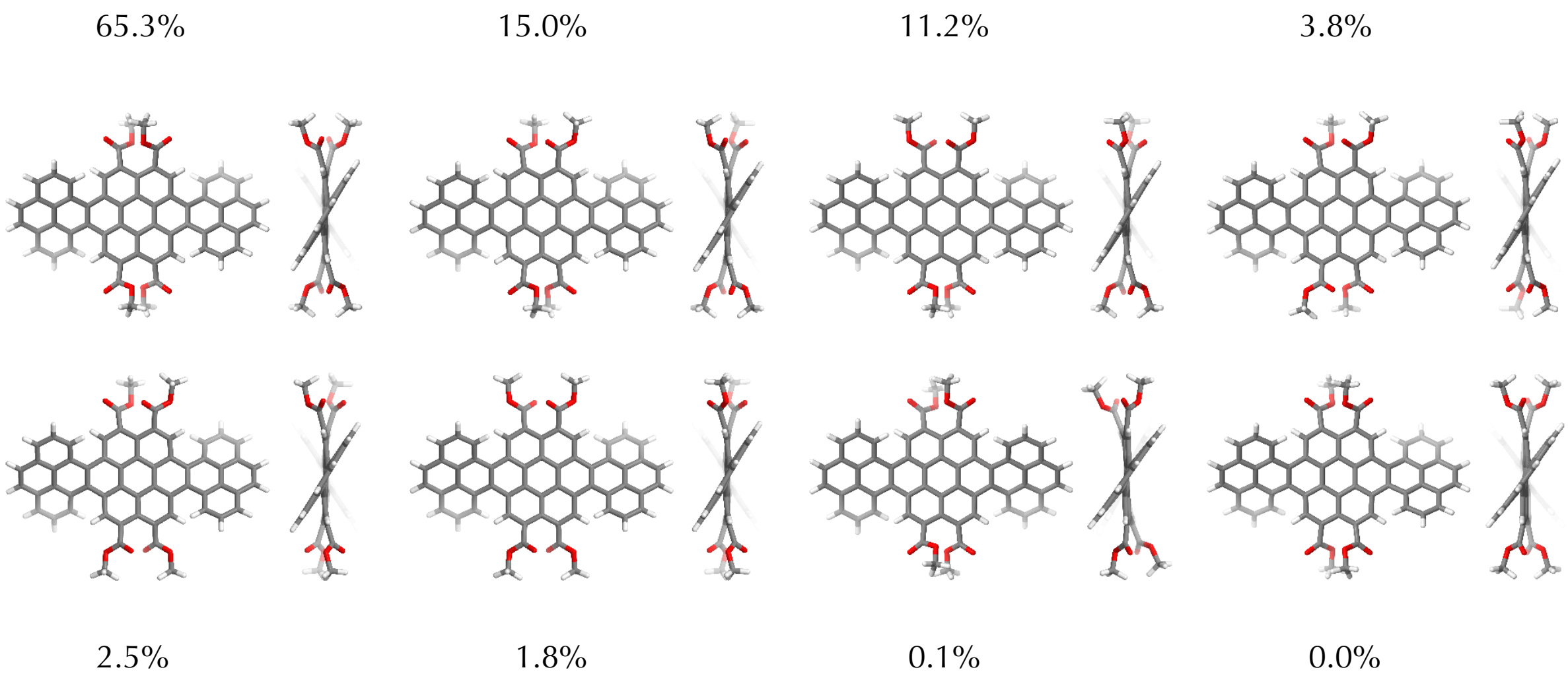

Figure S2. First minima of **NR-7-H** and relative populations computed at the M06-2X-6-31G(d,p) level, axial and longitudinal views.

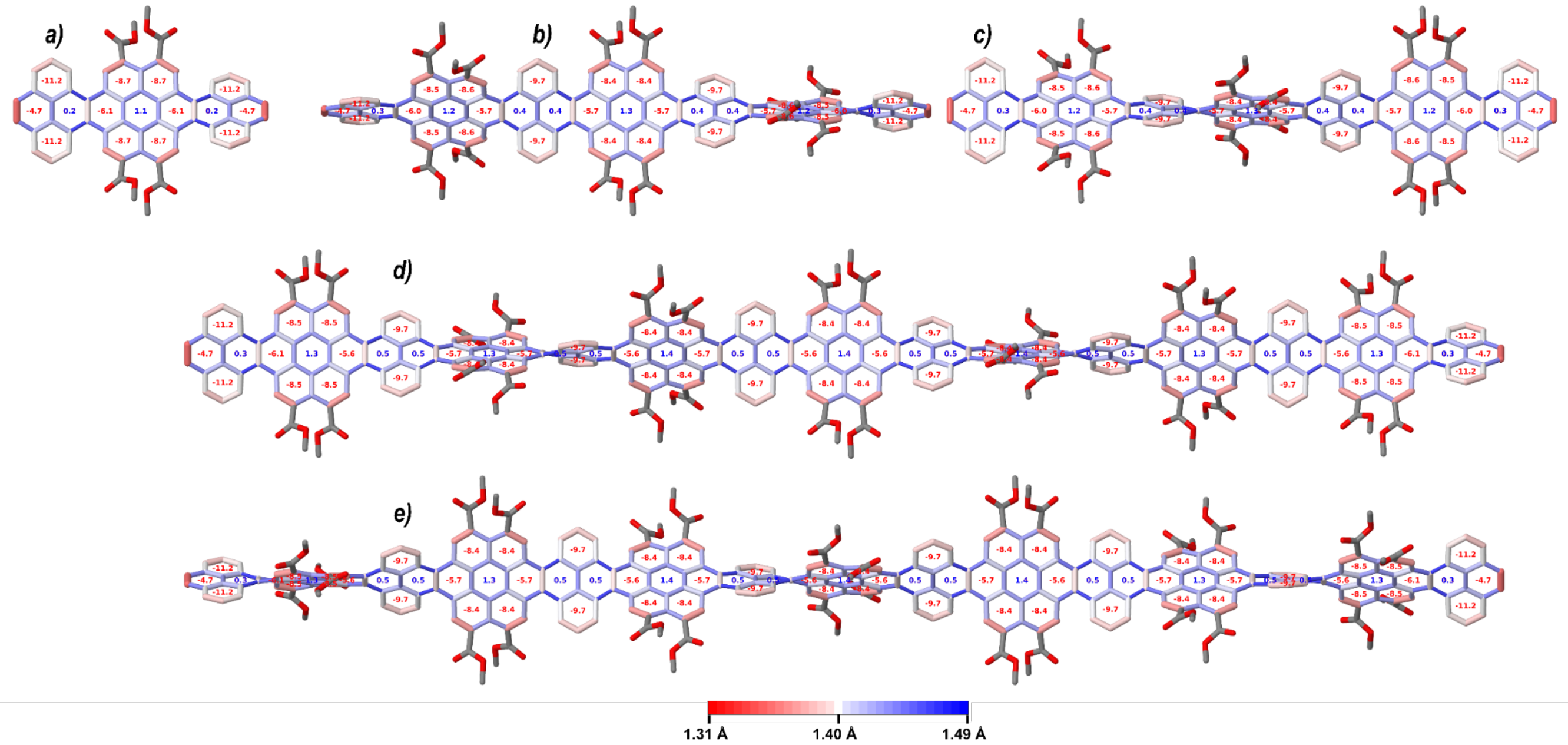

Figure S3. Bond length analysis and NICS(0) of **NR-7-H**, a), **NR-17-H**, two views, b) and c), and **NR-37-H**, two views, d) and e).

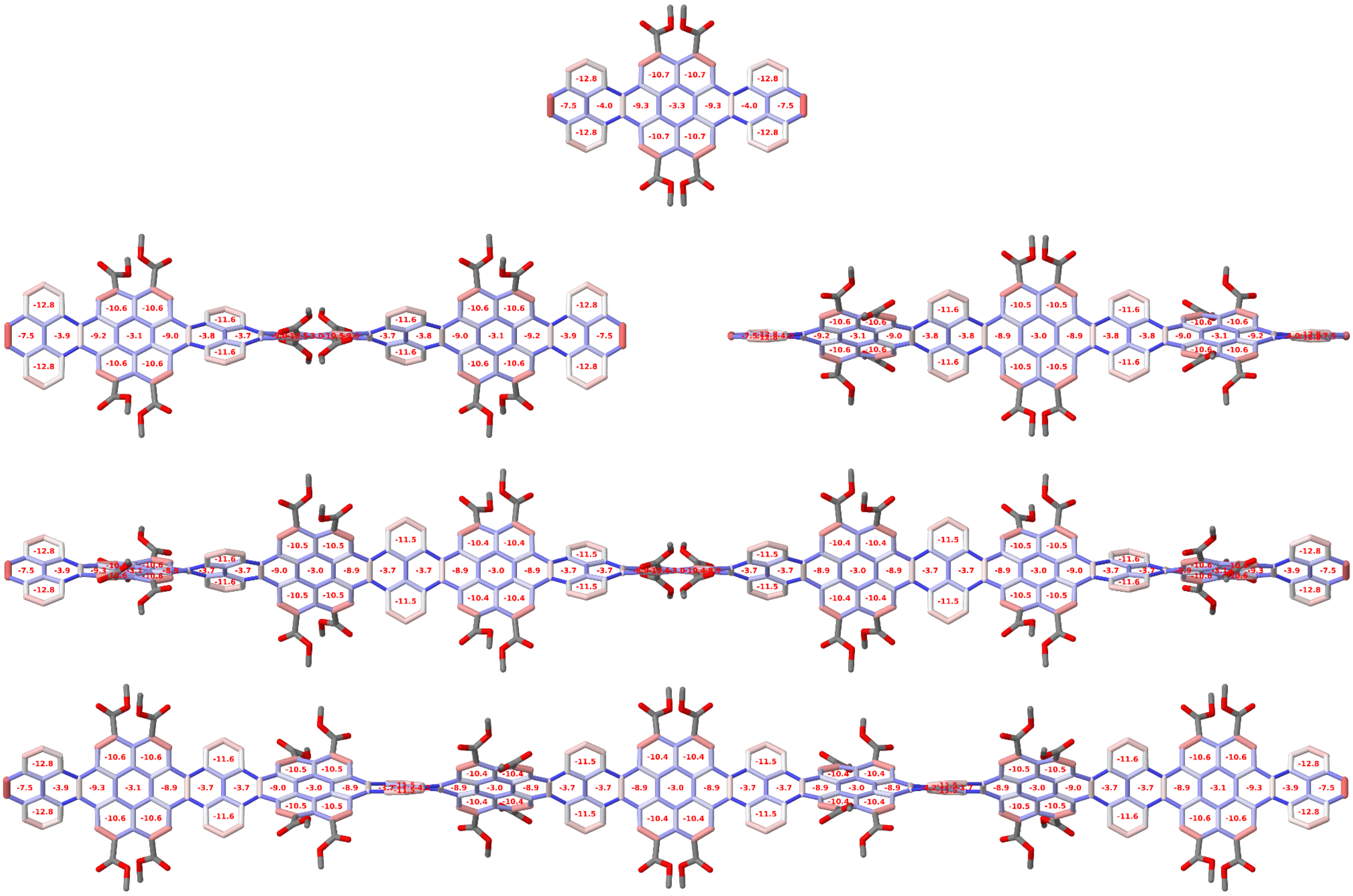

Figure S4. NICS(1) for **NR-7-H** (one orientation) and **NR-17-H** and **NR-37-H** (two alternative orientations).

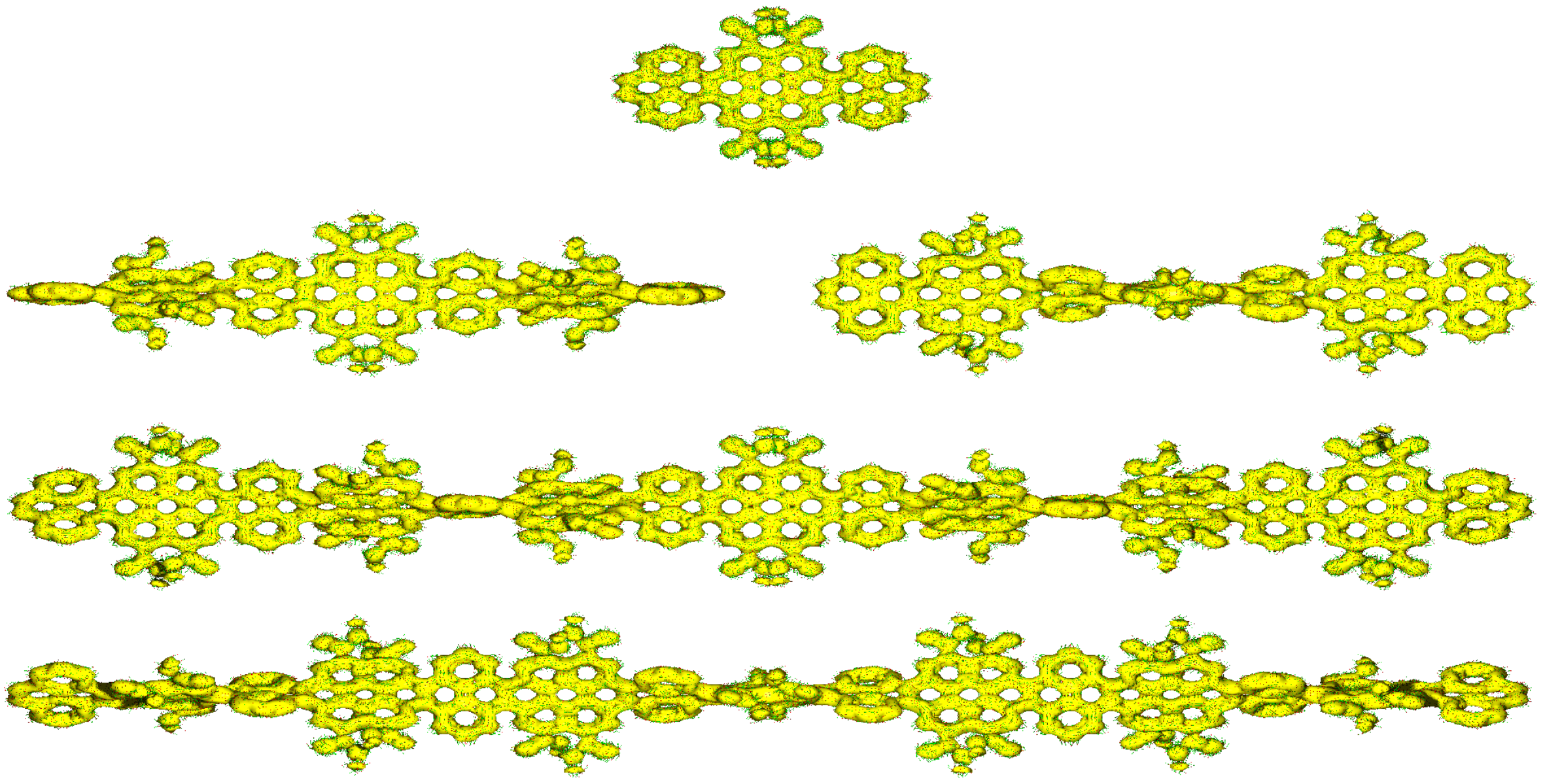

Figure S5. ACID analysis for **NR-7-H** (one orientation) and **NR-17-H** and **NR-37-H** (two alternative orientations).

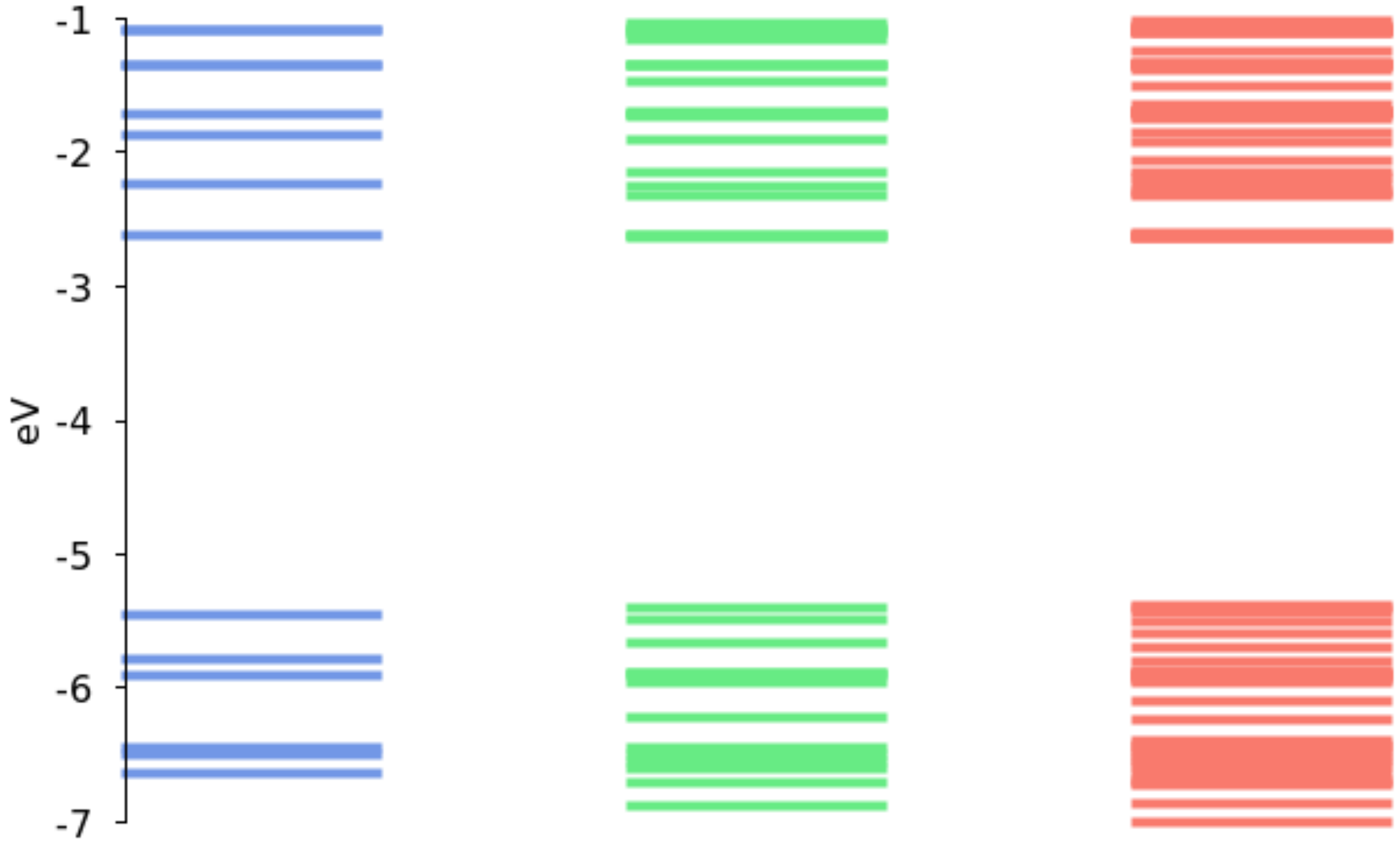

Figure S6. Frontier eigenvalues with the B3LYP Hamiltonian with the 6-311+G(2d,p) basis set in dichloromethane for **NR-7-H**, **NR-17-H** and **NR-37-H** on geometries optimized at the M06-2X-6-31G(d,p) level.

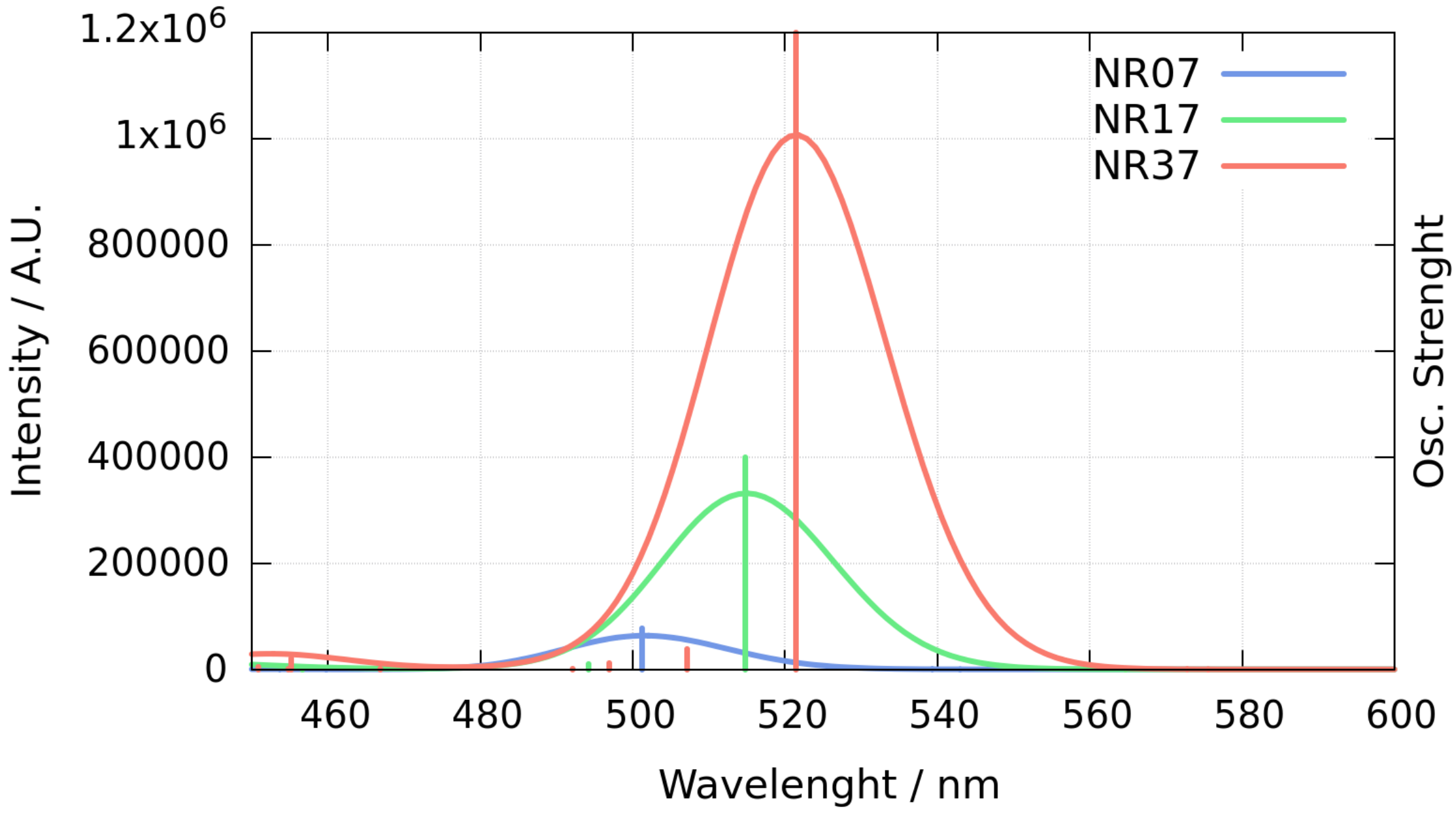

Figure S7. TD-DFT with the B3LYP Hamiltonian with the 6-311+G(2d, p) basis set in dichloromethane for **NR-7-H**, **NR-17-H** and **NR-37-H** on geometries optimized at the M06-2X-6-31G(d,p) level.

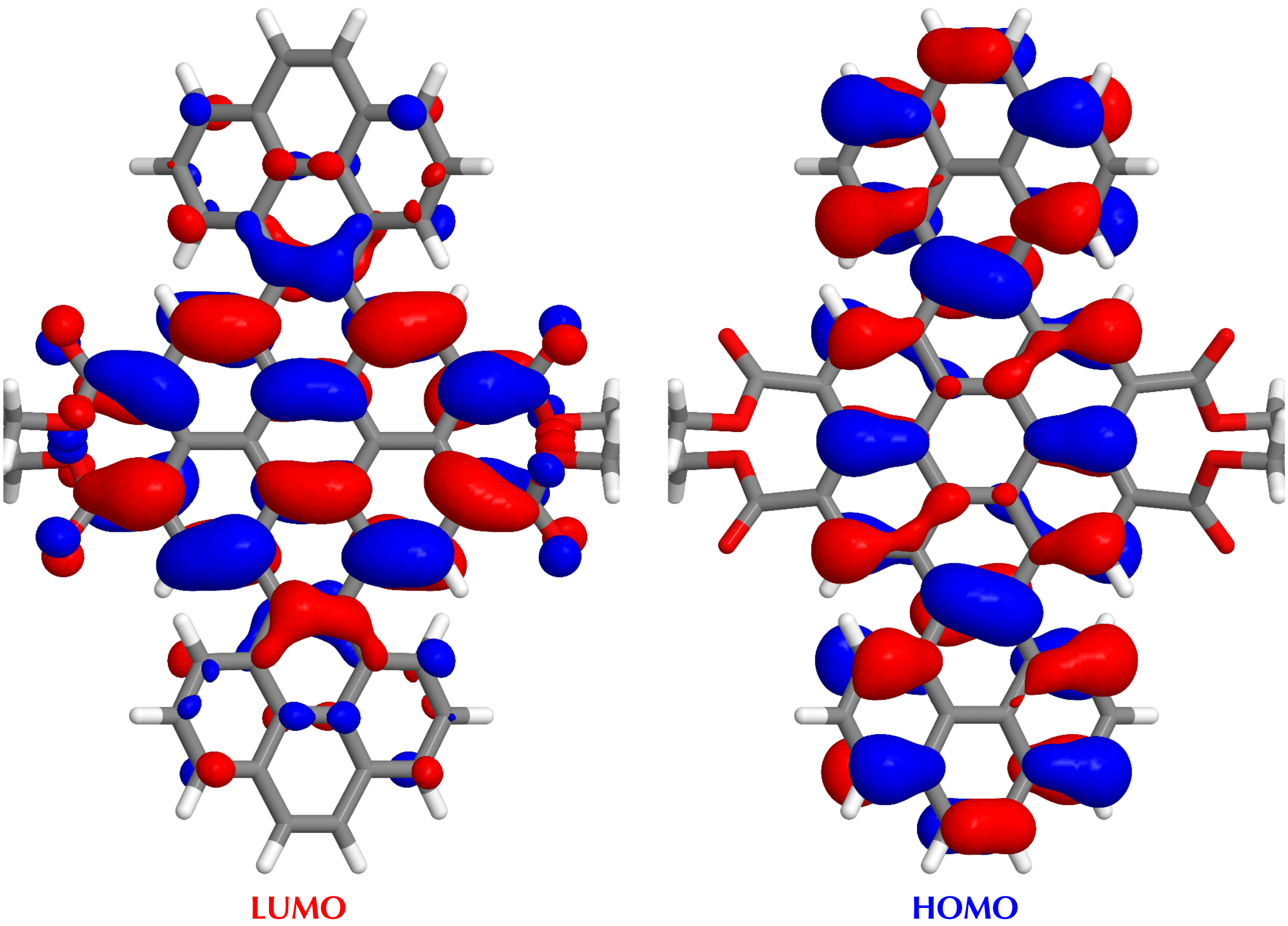

Figure S8. HOMO and LUMO of **NR-7-H** (isovalue 0.02).

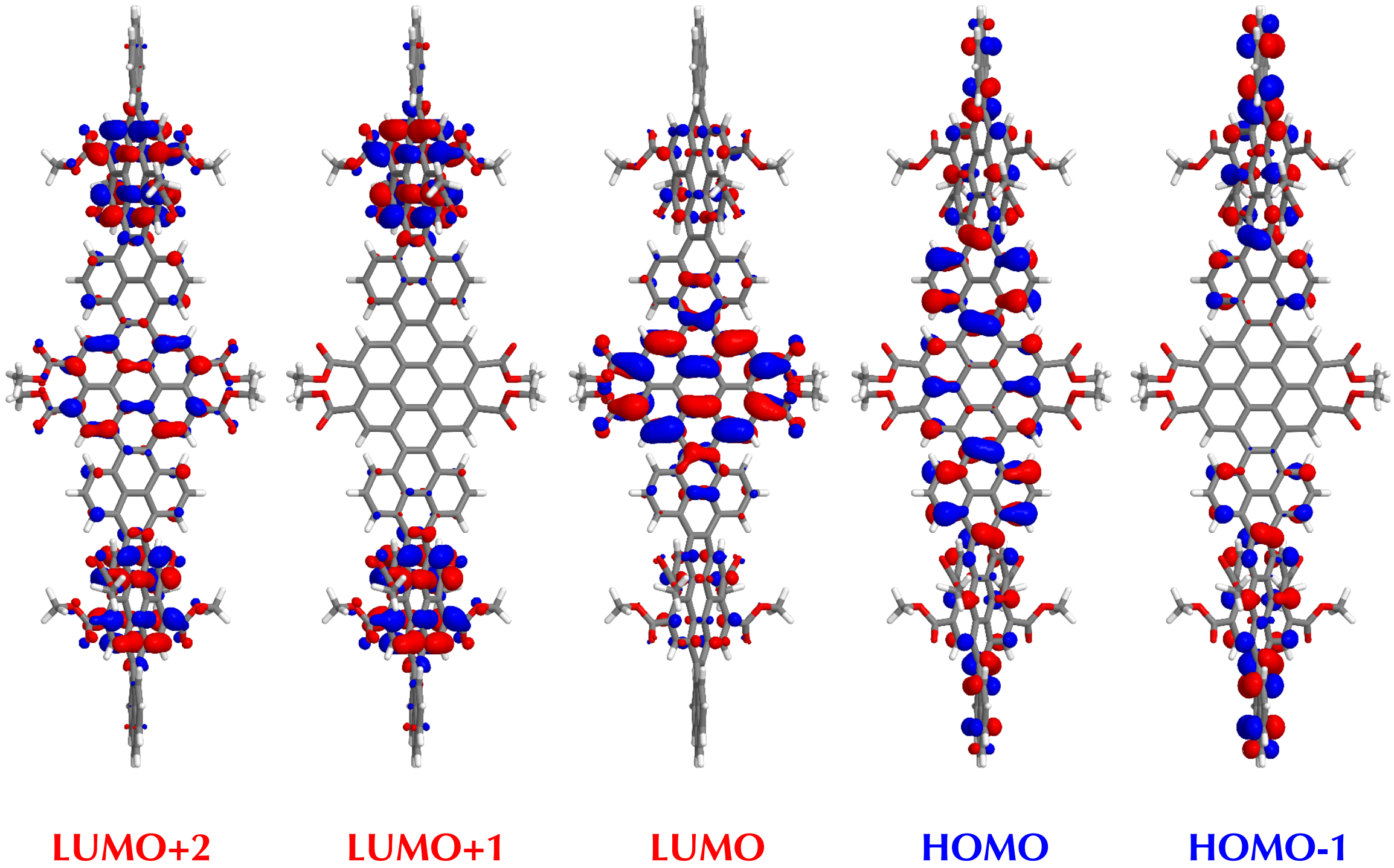

Figure S9. HOMO and LUMO of **NR-17-H** (isovalue 0.02).

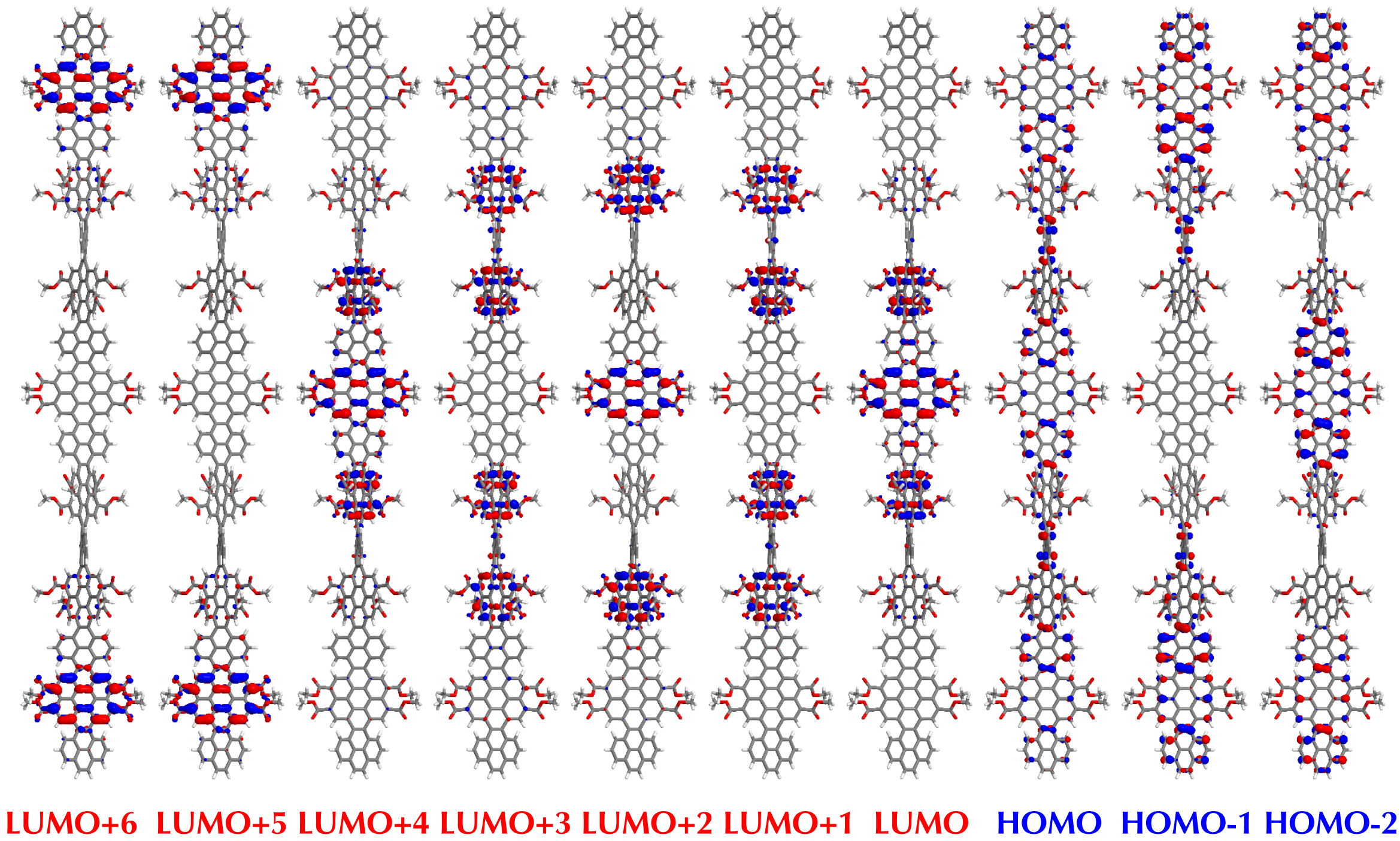

Figure S10. HOMO and LUMO of **NR-37-H** (isovalue 0.02).

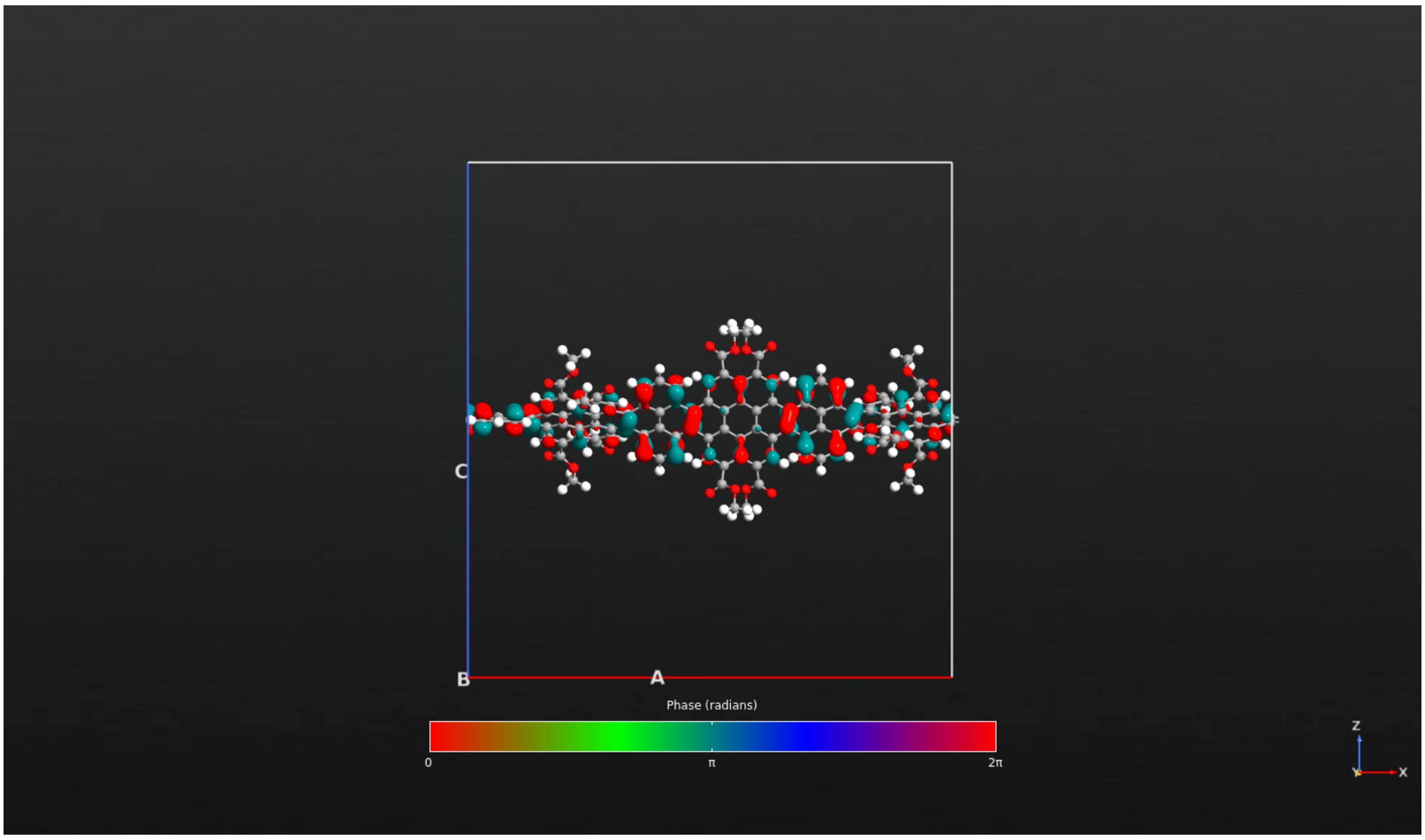

Figure S11. HOCO for periodic **NR-H** (isovalue 0.05).

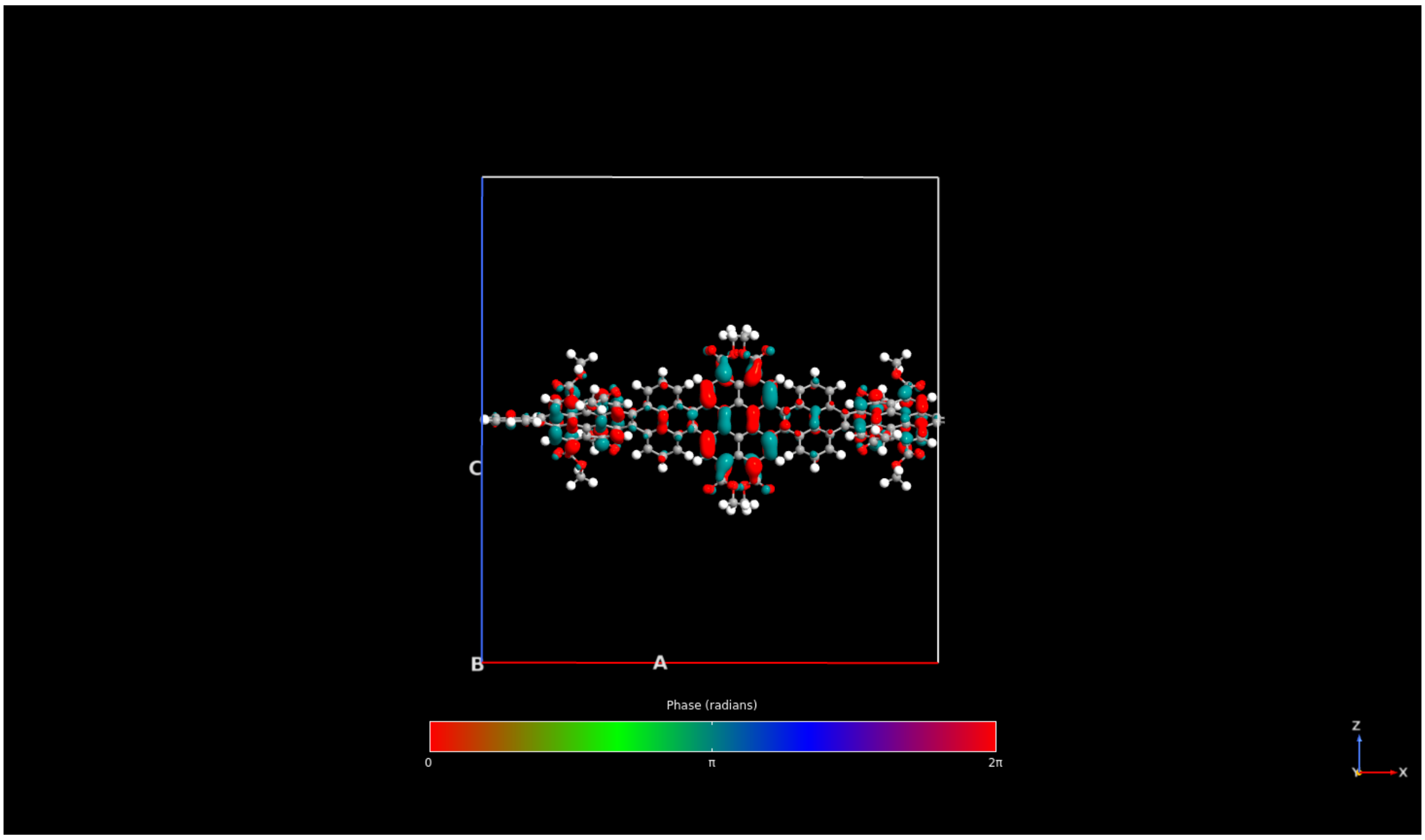

Figure S12. LUCO for periodic **NR-H** (isovalue 0.05).

Table S1. Frontier orbitals computed with the B3LYP Hamiltonian with the 6-31G(d,p) basis set in vacuum and with the 6-311+G(2d,p) basis set in dichloromethane for all NRs. All geometries were optimized at the M06-2X-6-31G(d,p) level. The colour code is: LUMOs, HOMOs and GAPs. All values in eV.

**B3LYP-6-31G(d,p)/M06-2X-6-31G(d,p)**																		
	L+8	L+7	L+6	L+5	L+4	L+3	L+2	L+1	**LUMO**	**HOMO**	H-1	H-2	H-3	H-4	H-5	H-6	H-7	**GAP**
**NR-7-H**	-0.51	-0.76	-0.77	-1.01	-1.06	-1.35	-1.62	-2	**-2.34**	**-5.25**	-5.6	-5.66	-6.25	-6.3	-6.42	-6.91	-6.91	**2.91**
**NR-17-H**	-1.42	-1.5	-1.69	-1.92	-2.04	-2.13	**-2.38**	**-2.39**	**-2.42**	**-5.25**	**-5.32**	-5.5	-5.69	-5.71	-5.72	-5.75	-6.05	**2.83**
**NR-37-H**	-2.15	-2.17	**-2.39**	**-2.39**	**-2.41**	**-2.42**	**-2.43**	**-2.44**	**-2.45**	**-5.25**	**-5.26**	**-5.31**	-5.37	-5.46	-5.56	-5.67	-5.7	**2.8**
**B3LYP-6-311+G(2d,p)-Dichloromethane/M06-2X-6-31G(d,p)**																		
**NR-7-H**	-0.84	-1.09	-1.09	-1.35	-1.35	-1.71	-1.87	-2.24	**-2.62**	**-5.45**	-5.79	-5.91	-6.45	-6.5	-6.63	-7.11	-7.17	**2.83**
**NR-17-H**	-1.72	-1.73	-1.91	-2.14	-2.25	-2.32	**-2.61**	**-2.62**	**-2.64**	**-5.4**	**-5.49**	-5.66	-5.88	-5.89	-5.91	-5.95	-6.21	**2.76**
**NR-37-H**	-2.31	-2.33	**-2.6**	**-2.61**	**-2.61**	**-2.62**	**-2.63**	**-2.64**	**-2.64**	**-5.38**	**-5.4**	**-5.44**	-5.51	-5.59	-5.69	-5.8	-5.87	**2.74**

Table S2. TD-DFT first six transitions with corresponding energy, wavelength, and oscillator strength computed at the B3LYP-6-31G(d,p) level on M06-2X-6-31G(d,p) geometries.

	Tr.	eV	nm	Osc.	MO contributions			
**NR-7-H**	**1**	**2.47**	**501**	**0.29**	**HOMO->LUMO(92%)**	H-2->L+1(7%)		
	2	2.80	443	0.00	H-2->LUMO(48%)	HOMO->L+1(50%)		
	3	2.84	436	0.00	H-1->LUMO(96%)			
	4	3.16	393	0.42	H-2->LUMO(48%)	HOMO->L+1(46%)	H-1->L+2(2%)	
	5	3.20	387	0.66	H-4->LUMO(15%)	H-2->L+1(77%)	HOMO->LUMO(7%)	
	6	3.21	386	0.01	H-1->L+1(87%)	H-3->LUMO(2%)	HOMO->L+2(8%)	
**NR-17-H**	**1**	**2.41**	**515**	**1.52**	H-1->L+1(16%)	**HOMO->LUMO(71%)**	H-2->LUMO(4%)	H-2->L+2(3%)
	2	2.47	501	0.00	H-1->LUMO(14%)	H-1->L+2(22%)	HOMO->L+1(54%)	H-3->L+4(2%)
	3	2.51	494	0.04	H-1->L+1(20%)	HOMO->L+2(56%)	H-6->L+3(4%)	H-4->L+4(2%)
	4	2.65	468	0.00	H-1->LUMO(73%)	H-1->L+2(17%)	H-5->LUMO(4%)	HOMO->L+1(3%)
	5	2.71	457	0.01	H-2->L+2(20%)	H-1->L+1(33%)	HOMO->L+2(29%)	H-5->L+1(3%)
	6	2.72	457	0.00	H-2->L+1(29%)	H-1->L+2(32%)	HOMO->L+1(30%)	H-1->LUMO(3%)
**NR-37-H**	**1**	**2.38**	**521**	**4.56**	H-2->LUMO(12%)	H-1->L+1(17%)	**HOMO->LUMO(36%)**	H-5->L+3(2%)
	2	2.41	514	0.00	H-1->LUMO(12%)	H-1->L+2(19%)	HOMO->L+1(32%)	H-4->L+1(3%)
	3	2.44	507	0.15	H-2->LUMO(15%)	H-1->L+3(16%)	HOMO->L+2(25%)	H-4->LUMO(3%)
	4	2.47	501	0.00	H-2->L+1(10%)	H-1->L+6(10%)	HOMO->L+3(19%)	H-3->LUMO(8%)
	5	2.49	497	0.04	H-2->L+6(10%)	H-1->L+5(22%)	HOMO->L+6(13%)	H-4->LUMO(4%)
	6	2.51	494	0.00	H-1->L+6(23%)	HOMO->L+3(14%)	HOMO->L+5(18%)	H-4->L+3(2%)

General Details

Reagents. All the commercial reagents utilized in the synthesis were used as received. Compound 2,7-di-*tert*-butyl-4-pinacolboronate-pyrene **A**[1] and tetrakis(2-ethylhexyl)-1,X-dibromoperylene-3,4,9,10-tetracarboxylate **B** (mixture of isomers 1,6 and 1,7)[2] were synthesized as reported previously. 3,4,7,8-Tetramethyl-1,10-phenanthroline = Me_4phen. dtbpy = 4,4′-Di-tert-butyl-2,2′-dipyridyl.

Synthesis. All the reactions were performed in an oven-dried round-bottom flask or Schlenk tube. The required high temperature for the reactions was achieved using an oil-bath or an aluminium heating block. The reaction progress was regularly monitored by thin-layer chromatography on TLC plates for which visualization was realized either by visual observation with naked eye or by irradiation with UV lamp.

Purification. The purification of the products was performed by silica-gel column chromatography under ambient conditions. The sorbent for the column chromatography (silica gel 60, 0.04–0.06 mm, 230–400 mesh) and the TLC plates were purchased from commercial suppliers. The monitoring of the purification process was performed by thin-layer chromatography on TLC plates.

Characterization. The NMR spectra were recorded with 400 MHz pulsed Fourier transform NMR spectrometer in deuterated solvents at room temperature. The chemical shift values are given in ppm and *J* values in Hz. High-resolution mass spectra of all the compounds were recorded on Bruker Daltonics-Autoflex MALDITOF mass spectrometer by Dr. Estibaliz Gonzalez de San Roman Martin.

Absorption and fluorescence spectroscopy. The absorption spectra were recorded with a double beam UV/vis/NIR spectrophotometer (PerkinElmer – Lambda 950) and the emission spectra were collected on a fluorescence spectrometer (PerkinElmer – LS 55). Fluorescence quantum yields were determined by the comparative method using rhodamine 6G (Φ = 0.94 in ethanol)[3] as a standard.

Cyclic voltammetry. Electrochemical behaviour of the compounds was studied using cyclic voltammetry in a three-electrode single-compartment cell consisting of a glassy carbon working electrode, silver wire as the reference electrode, and a platinum wire as the counter electrode with ferrocene (Fc) as an internal reference (0.3 mM). The cell was connected to the computer controlled potentiostat (Princeton Applied Research-PARSTAT 2273). The measurements were carried out under Ar atmosphere in anhydrous CH_2Cl_2 usingtetrabutylammonium hexafluorophosphate (0.1 M) as the supporting electrolyte. The concentration of the prepared samples was in the range of 0.1–0.5 mM.

Time-resolved terahertz spectroscopy (TRTS). TRTS was used to characterize the microscopic charge transport properties of GNRs of different lengths. GNR films were prepared by drop-casting toluene dispersions of the GNR powers onto fused silica substrates to form thin films (thickness of ~500 nm). TRTS measurements were carried out using a commercial regenerative Ti:sapphire amplifier system (800 nm center wavelength, 1 kHz repetition rate, ~50 fs pulse duration). The laser output was split into three beams to drive THz generation, THz detection, and optical excitation. Single-cycle THz pulses were generated via optical rectification in a <110>-oriented ZnTe crystal and subsequently detected using free-space electro-optic sampling in a second ZnTe crystal.[20] For sample excitation, 400 nm pump pulses were generated by frequency doubling the fundamental 800 nm output in a *β*-barium borate (BBO) crystal. The excitation fluence was used at ~1×10^{15} photons·cm^{-2}·$pulse^{-1}$. To minimize THz absorption by atmospheric water vapor, all measurements were conducted at room temperature within a dry nitrogen-purged environment.

Synthetic procedures

Synthesis of NR-7

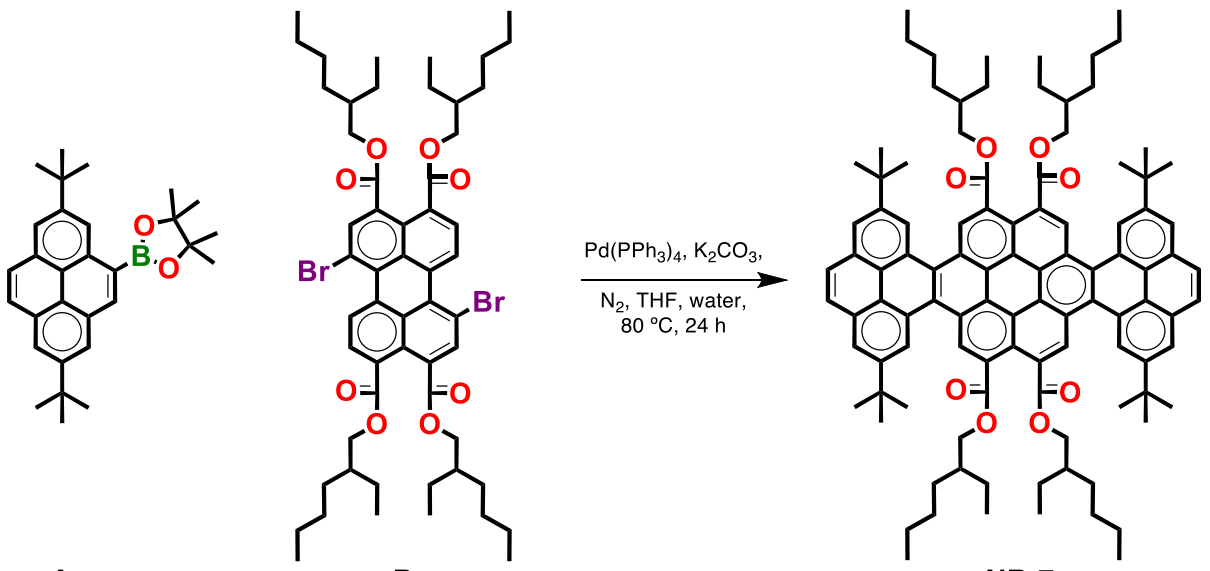

A B NR-7
1,6- & 1,7- regioisomeric mixture

A Schlenk flask was charged with **A** (50 mg, 0.11 mmol), **B** (40 mg, 0.04 mmol), $Pd(PPh_3)_4$ (9 mg, 0.01 mmol), and K_2CO_3 (107 mg, 0.77 mmol). The reaction mixture was deaerated using vacuum and backfilled with nitrogen three times. Subsequently, THF (40 mL) and water (10 mL) were bubbled with N_2 and added to the reaction. Then, the mixture was refluxed for 24 h under an inert atmosphere. After being cool to room temperature, dichloromethane was added, and the organic phase was separated using a separatory funnel and washed with water. The organic phase was dried over sodium sulphate, concentrated, and purified by column chromatography using a mixture of hexane/dichloromethane (3:2) as eluent to afford the desired product (12 mg, 20%). NMR spectra match with the literature.[2]

Synthesis of NR-7-Bpin

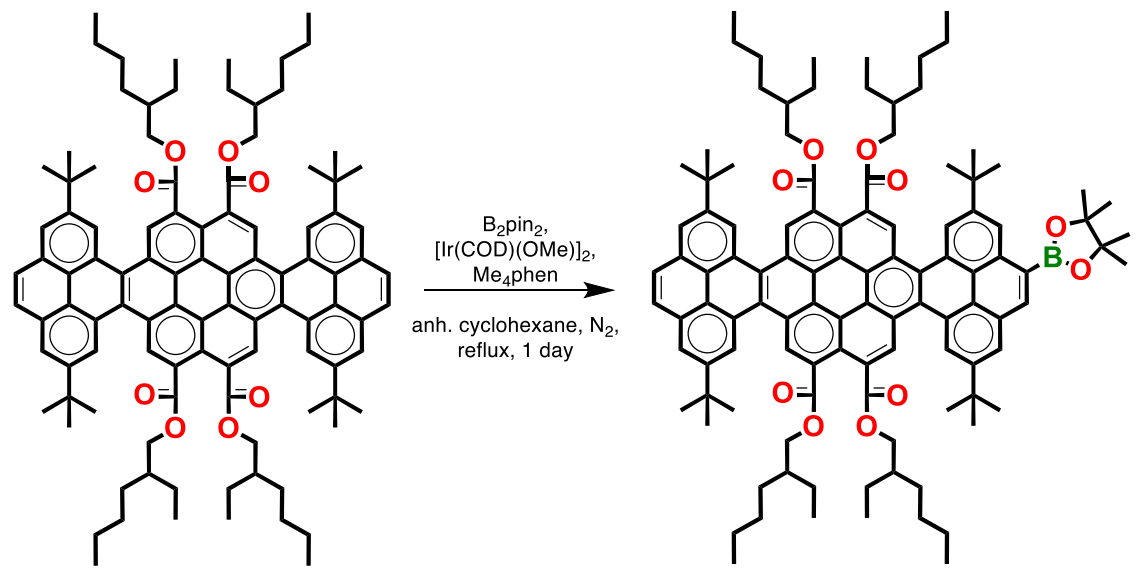

NR-7 NR-7-Bpin

A Schlenk flask was charged with $[Ir(OMe)COD]_2$ (58 mg, 0.1 mmol), Me_4phen (41 mg, 0.2 mmol), B_2pin_2 (220 mg, 0.9 mmol), **NR-7** (650 mg, 0.4 mmol), and a magnetic stirred. Then, the reaction mixture was deaerated using vacuum and backfilled with nitrogen three times. Anhydrous cyclohexane (40 mL) was added by syringe, and the mixture was heated at reflux for 1 d. After being cooled to room temperature, the reaction mixture was poured in water, and the crude product was extracted with dichloromethane. The combined organic phase was dried over sodium sulphate, concentrated, and purified by column chromatography using a mixture of hexane/dichloromethane (3:2) as eluent to afford the desired product (282 mg, 40%). ^{1}H NMR (400 MHz, $CDCl_3$) δ 10.48 (s, 3H), 10.45 (s, 1H), 9.54–9.45 (m, 5H), 8.89 (s, 1H), 8.51 (s, 1H), 8.45 (s, 1H), 8.25 (s, 2H), 4.54–4.42 (m, 8H), 1.97 (p, *J* = 6.1 Hz, 4H), 1.78 (s, 9H), 1.78 (s, 18H), 1.76 (s, 9H), 1.62 (s, 12H), 1.58–1.17 (m, 24H), 0.93 (t, *J* = 7.4 Hz, 12H), 0.78 (t, *J* = 6.6 Hz, 12H). ^{13}C NMR (101 MHz, CDCl3) δ 169.16, 149.40, 149.22, 138.89, 133.68, 131.31, 130.87, 130.64, 130.42, 129.28, 128.86, 128.62, 128.48, 128.36, 128.07, 127.85, 127.19, 126.49, 126.31, 126.20, 125.62, 124.83, 124.36, 124.00, 123.86, 123.61, 123.49, 84.19, 68.70, 68.62, 38.79, 36.01, 35.84, 35.79, 32.24, 32.19, 30.29, 28.97, 25.36, 23.75, 23.09, 14.11, 10.87. MS (MALDI): m/z calc. for $C_{110}H_{131}BO_{10}$ [M^+]: 1621.987, found 1621.993.

Synthesis of NR-17

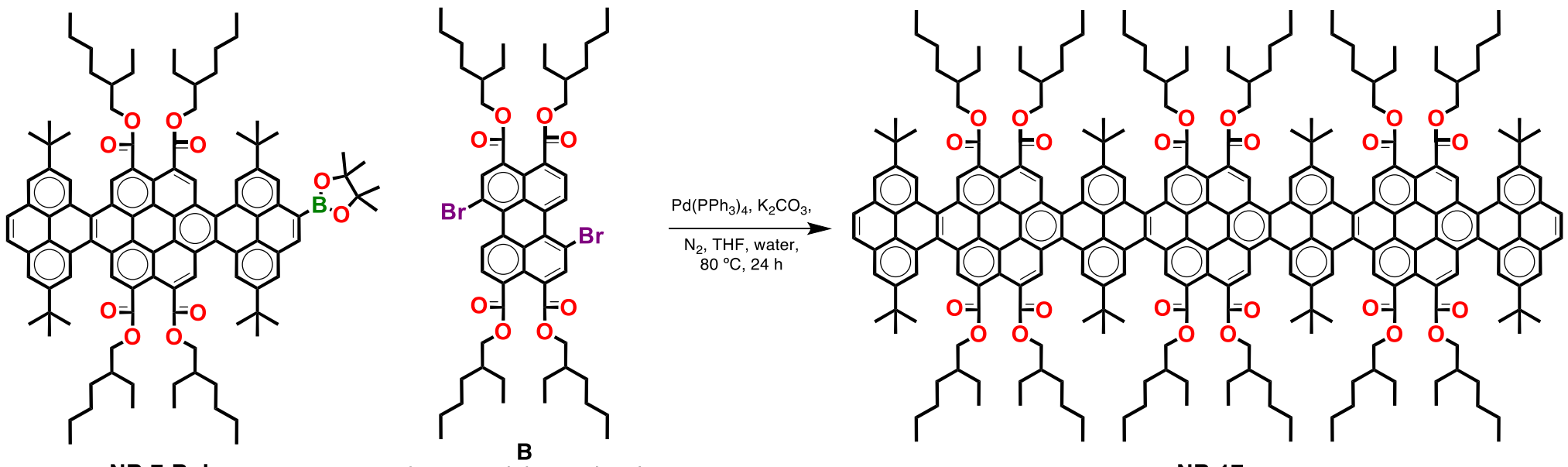

A Schlenk flask was charged with **NR-7-Bpin** (200 mg, 0.12 mmol), **B** (32 mg, 0.03 mmol), $Pd(PPh_3)_4$ (7.1 mg, 0.006 mmol), and K_2CO_3 (85 mg, 0.62 mmol). The reaction mixture was deaerated using vacuum and backfilled with nitrogen three times. Subsequently, THF (32 mL) and water (6.4 mL) were bubbled with N_2 and added to the reaction. Then, the mixture was refluxed for 24 h under an inert atmosphere. After being cool to room temperature, dichloromethane was added and the organic phase was separated using a separatory funnel and washed with water. The organic phase was dried over sodium sulphate, concentrated, and purified by column chromatography using a mixture of hexane/dichloromethane (1:1) as eluent to afford the desired product (42 mg, 30%). ^{1}H NMR (400 MHz, $CDCl_3$) δ 10.62 (s, 1H), 10.58 (s, 1H), 10.54 (s, 1H), 9.78 (s, 2H), 9.54 (s, 1H), 8.47 (s, 1H), 8.28 (s, 1H), 4.60–4.44 (m, 6H), 2.07–1.92 (m, 12H), 1.80 (s, 9H), 1.61–1.16 (m, 24H), 1.01–0.91 (m, 9H), 0.84–0.72 (m, 9H). ^{13}C NMR (101 MHz, CDCl3) ^{13}C NMR (101 MHz, $CDCl_3$) δ 169.17, 150.33, 149.49, 131.37, 130.89, 130.75, 130.59, 128.97, 128.87, 128.79, 128.73, 128.48, 128.08, 127.90, 126.97, 126.63, 126.49, 126.38, 125.82, 125.74, 124.35, 124.27, 124.01, 123.83, 123.67, 123.63, 68.86, 68.79, 38.85, 36.48, 35.87, 32.44, 32.27, 30.31, 29.85, 29.00, 23.78, 23.12, 14.12, 10.88. MS (MALDI): m/z calc. for $C_{264}H_{308}O_{24}$ $[M+H]^+$: 3863.294, found 3863.305.

Synthesis of NR-17-Bpin

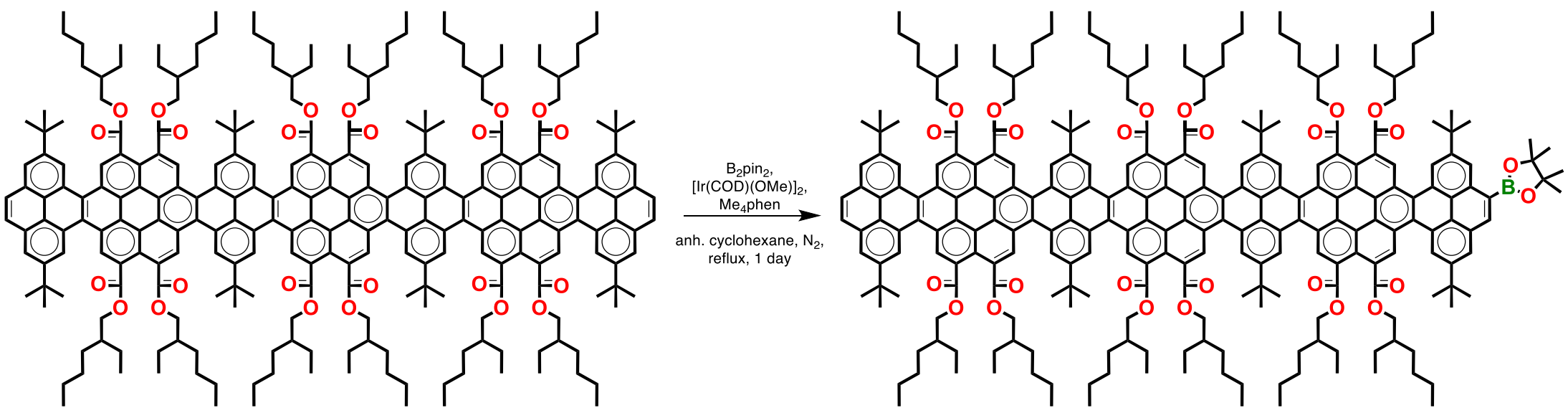

A flame-dried flask was charged with $[Ir(OMe)COD]_2$ (3.4 mg, 0.005 mmol), Me_4phen (2.4 mg, 0.01 mmol), B_2pin_2 (32.8 mg, 0.13 mmol), **NR-17** (100 mg, 0.03 mmol), and a magnetic stirred. Then, the reaction mixture was deaerated using vacuum and backfilled with nitrogen three times. Anhydrous cyclohexane (40 mL) was added by syringe, and the mixture was heated at reflux for 1 d. After being cooled to room temperature, the reaction mixture was poured in water, and the crude product was extracted with dichloromethane. The combined organic phase was dried over sodium sulphate, concentrated, and purified by column chromatography using a mixture of hexane/dichloromethane (1:1) as eluent to afford the desired product (99 mg, 97%). ^{1}H NMR (400 MHz, $CDCl_3$) δ 10.62 (s, 4H), 10.59 (s, 2H), 10.58 (s, 2H), 10.54 (s, 3H), 10.51 (s, 1H), 9.78 (s, 8H), 9.62–9.46 (m, 5H), 8.91 (s, 1H), 8.54 (s, 1H), 8.48 (s, 2H), 8.28 (s, 2H), 4.92–4.30 (m, 24H), 2.08–1.95 (m, 44H), 1.80 (s, 27H), 1.78 (s, 9H), 1.63 (s, 12H), 1.55–1.17 (m, 96H), 1.02–0.90 (m, 36H), 0.89–0.72 (m, 36H). ^{13}C NMR (101 MHz, $CDCl_3$) δ 169.18, 150.32, 149.49, 149.29, 131.37, 130.76, 130.57, 128.96, 128.86, 128.48, 128.07, 127.91, 126.97, 126.63, 126.49, 126.38, 125.74, 124.94, 124.35, 124.01, 123.83, 123.67, 84.23, 68.81, 38.85, 36.48, 32.44, 32.27, 32.22, 30.31, 29.86, 29.00, 25.38, 23.77, 23.12, 14.12, 10.88. MS (MALDI): m/z calc. for $C_{270}H_{319}BO_{26}$ $[M^+]$: 3990.383, found 3990.575.

Synthesis of NR-37

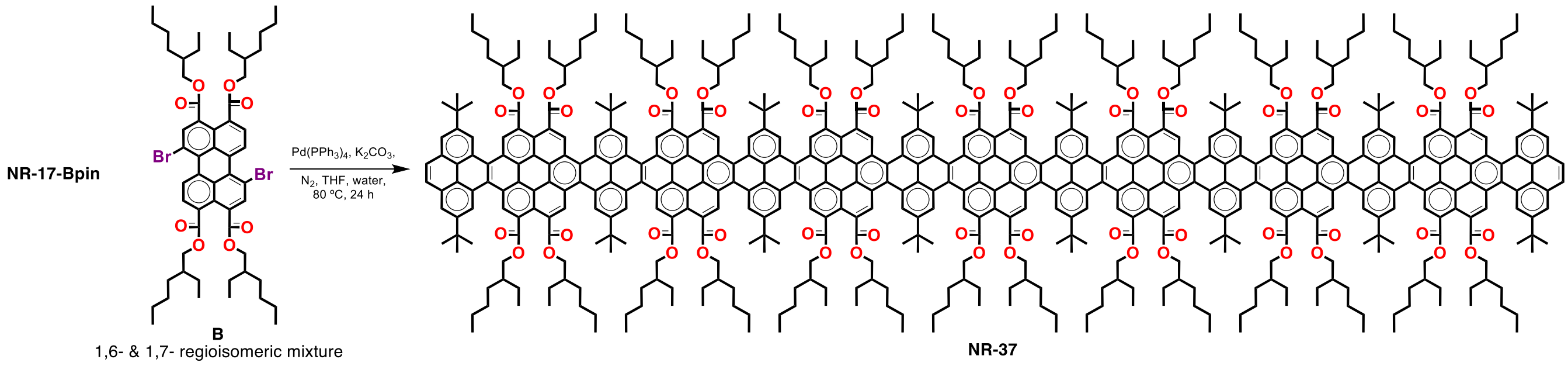

A Schlenk flask was charged with **B** (8 mg, 0.01 mmol), **NR-17-Bpin** (130 mg, 0.03 mmol), $Pd(PPh_3)_4$ (2 mg, 0.002 mmol), and K_2CO_3 (21 mg, 0.15 mmol). The reaction mixture was deaerated using vacuum and backfilled with nitrogen three times. Subsequently, THF (8 mL) and water (1.6 mL) were bubbled with N_2 and added to the reaction. Then, the mixture was refluxed for 24 h under an inert atmosphere. After being cool to room temperature, dichloromethane was added, and the organic phase was separated using a separatory funnel and washed with water. The organic phase was dried over sodium sulphate, concentrated, and purified by column chromatography using a mixture of hexane/dichloromethane (1:4) as eluent to afford the desired product (50 mg, 75%). ^{1}H NMR (400 MHz, $CDCl_3$) δ 10.68–10.53 (m, 7H), 9.78 (s, 6H), 9.54 (s, 1H), 8.48 (s, 1H), 8.28 (s, 1H), 4.53 (s, br, 14H), 2.02 (s, 3H), 2.00 (s, 4H), 1.87 (dd, *J* = 16.4, 11.1 Hz, 1H), 1.80 (s, 4H), 1.50–1.18 (m, 4H), 1.06–0.90 (m, 3H), 0.89–0.71 (m, 5H). ^{13}C NMR (101 MHz, $CDCl_3$) δ 169.17, 150.35, 149.50, 130.76, 128.98, 128.86, 128.49, 126.49, 125.80, 124.36, 123.85, 77.47, 77.16, 76.84, 68.85, 38.86, 36.49, 32.45, 32.26, 30.32, 29.85, 29.01, 23.79, 23.13, 14.13, 10.89. MS (MALDI): m/z calc. for $C_{584}H_{684}O_{56}$: 8599.088, found 8599.560.

Table S3. Optical and Electronic Properties of NRs in Solution

Compound	λ_{abs} (ε M^{-1} cm^{-1})	λ_{em} (nm)	Φ_f [a]	$\varepsilon\Phi$ (M^{-1} cm^{-1})	E_{1ox}	E_{1red}	E_{2red}	$E_{g(opt)}$ [b] (eV)	E_{LUMO} [c] (eV)	E_{HOMO} [d] (eV)	$E_{HOMO-LUMO}$ (eV)
**NR-7**	391 (144000), 418 (59400), 496 (13500), 530 (31300)	546, 586	0.62	8.9×10^4	0.76	−1.81	−2.04	2.24	−2.99	−5.56	2.64
**NR-17**	400 (380000), 425 (340000), 497 (65500), 536 (194000)	550, 589	0.40	1.5×10^5	0.79	−1.82	−2.07	2.20	−2.98	−5.59	2.61
**NR-37**	401 (586000), 425 (560000), 497 (100000), 536 (310000)	550, 589	0.51	3.0×10^5	0.78	−1.82	−2.08	2.20	−2.98	−5.58	2.60

The absorption and emission were measured in dichloromethane. The redox potentials (V vs Fc/Fc^+) obtained by cyclic voltammetry in dichloromethane using 0.1M $n Bu_4NPF_6$ as the supporting electrolyte (Scan rate = 100 mV/s). The potentials are reported as $E_{1/2} = (E_p^a + E_p^c)/2$ and quoted to the nearest 0.01 V. [a]Rhodamine 6G was used as a standard. [b]Optical band gap calculated using equation $E_g = hc/\lambda_{ae} \approx 1240/\lambda ae$ (nm); where λ_{ae} denotes the absorption edge wavelength in nm, obtained from offset wavelength derived from the lowest energy absorption band in dichloromethane. [c]The LUMO levels were estimated from the onset of the first reduction potential $E_{LUMO} = -(E_{1red(ONSET)} + 4.8eV)$. [d]The HOMO levels were estimated from the onset of the first reduction potential $E_{HOMO} = -(E_{1ox(ONSET)} + 4.8eV)$.

NMR Spectra

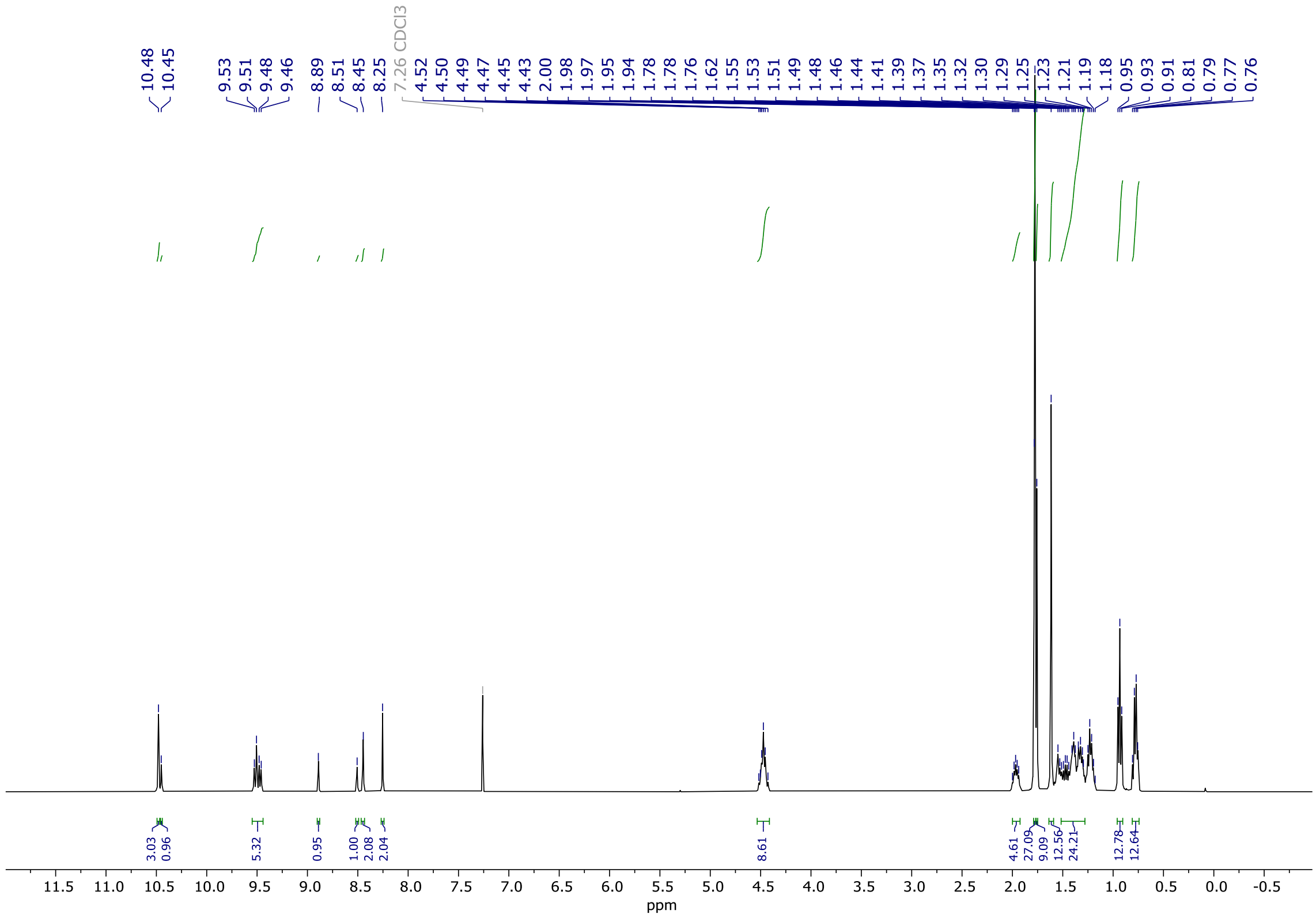

Figure S13. ^{1}H NMR (400 MHz, $CDCl_3$) of **NR-7-Bpin**.

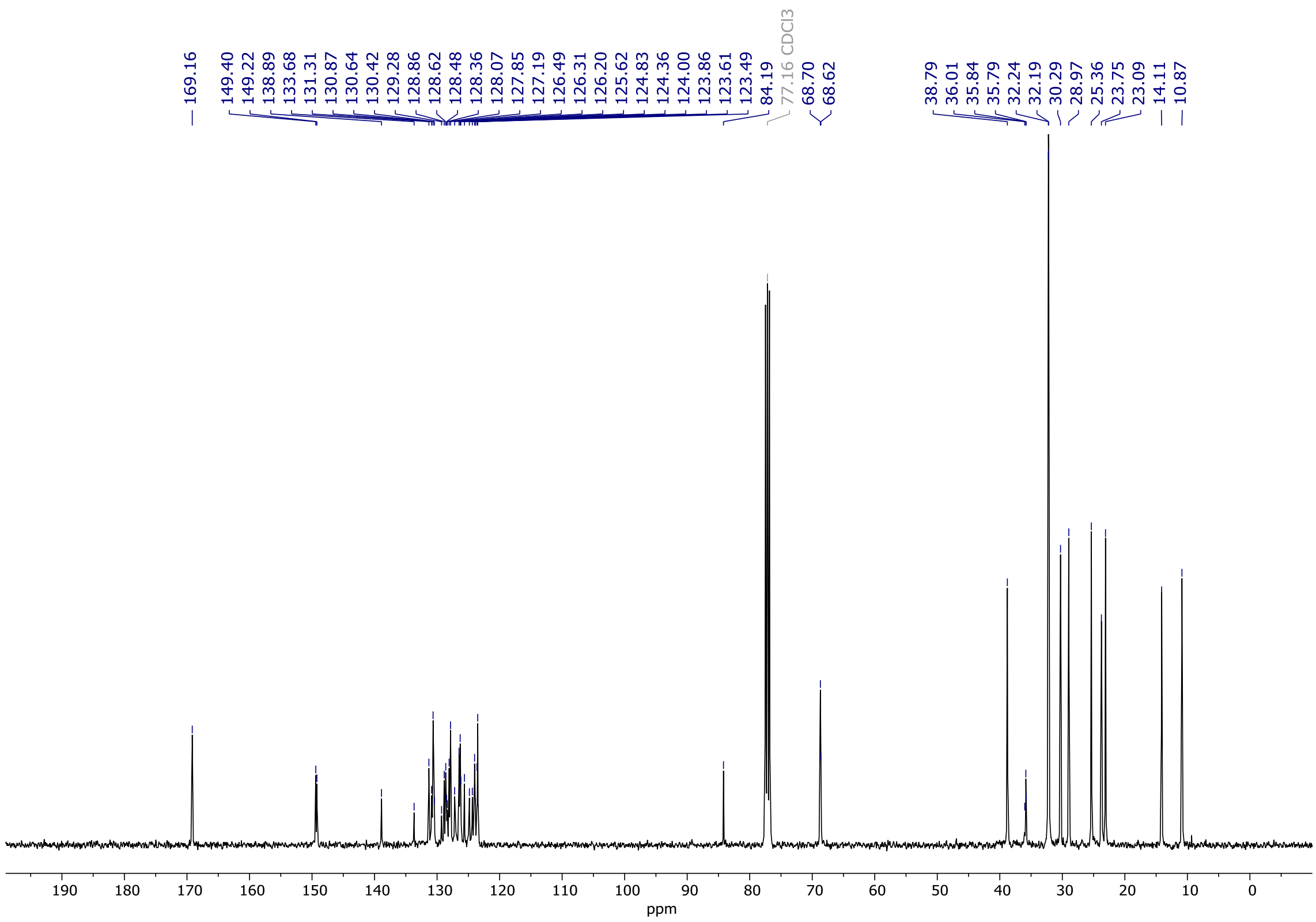

Figure S14. ^{13}C NMR (101 MHz, $CDCl_3$) of **NR-7-Bpin**.

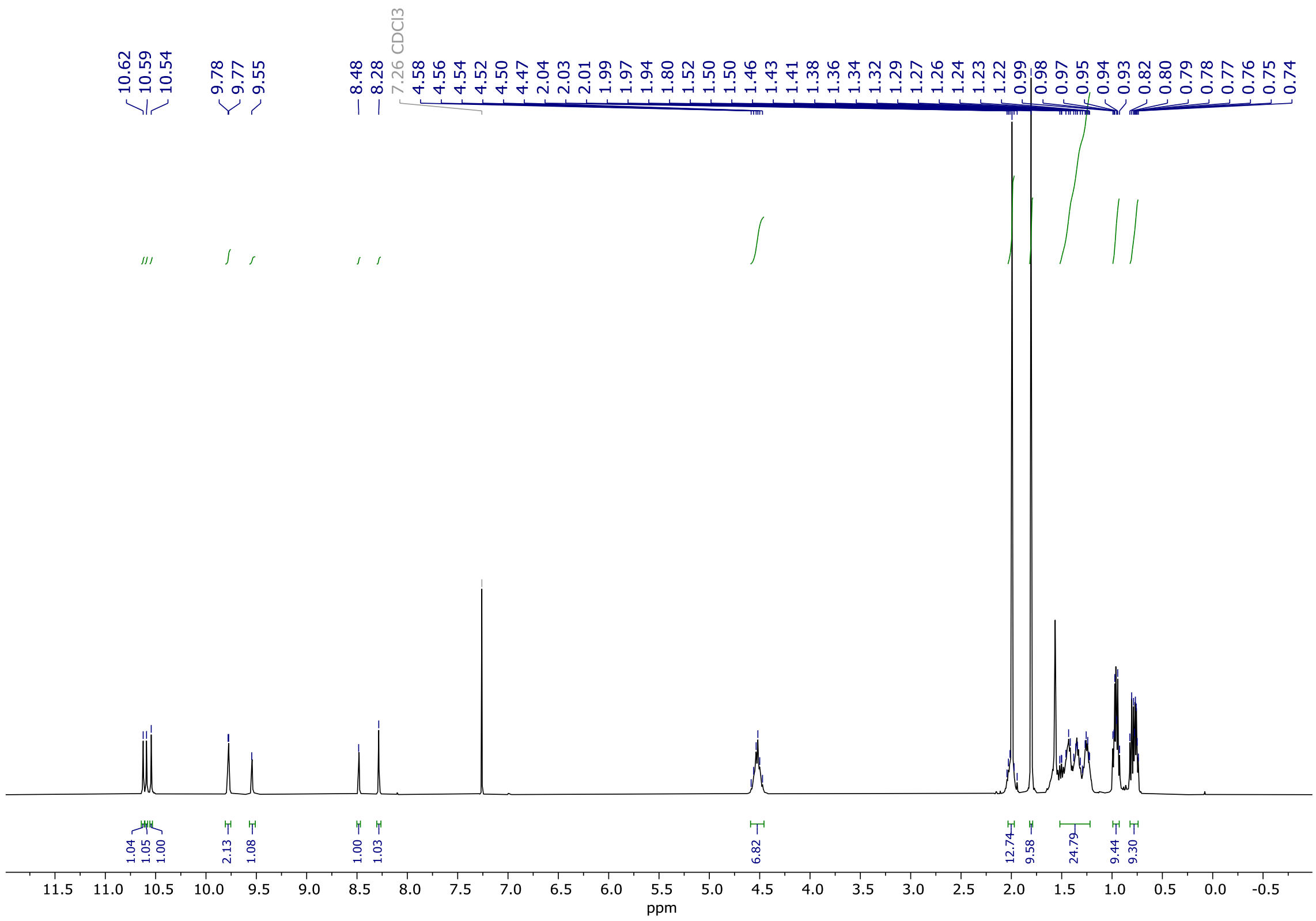

Figure S15. ^{1}H NMR (400 MHz, $CDCl_3$) of **NR-17**.

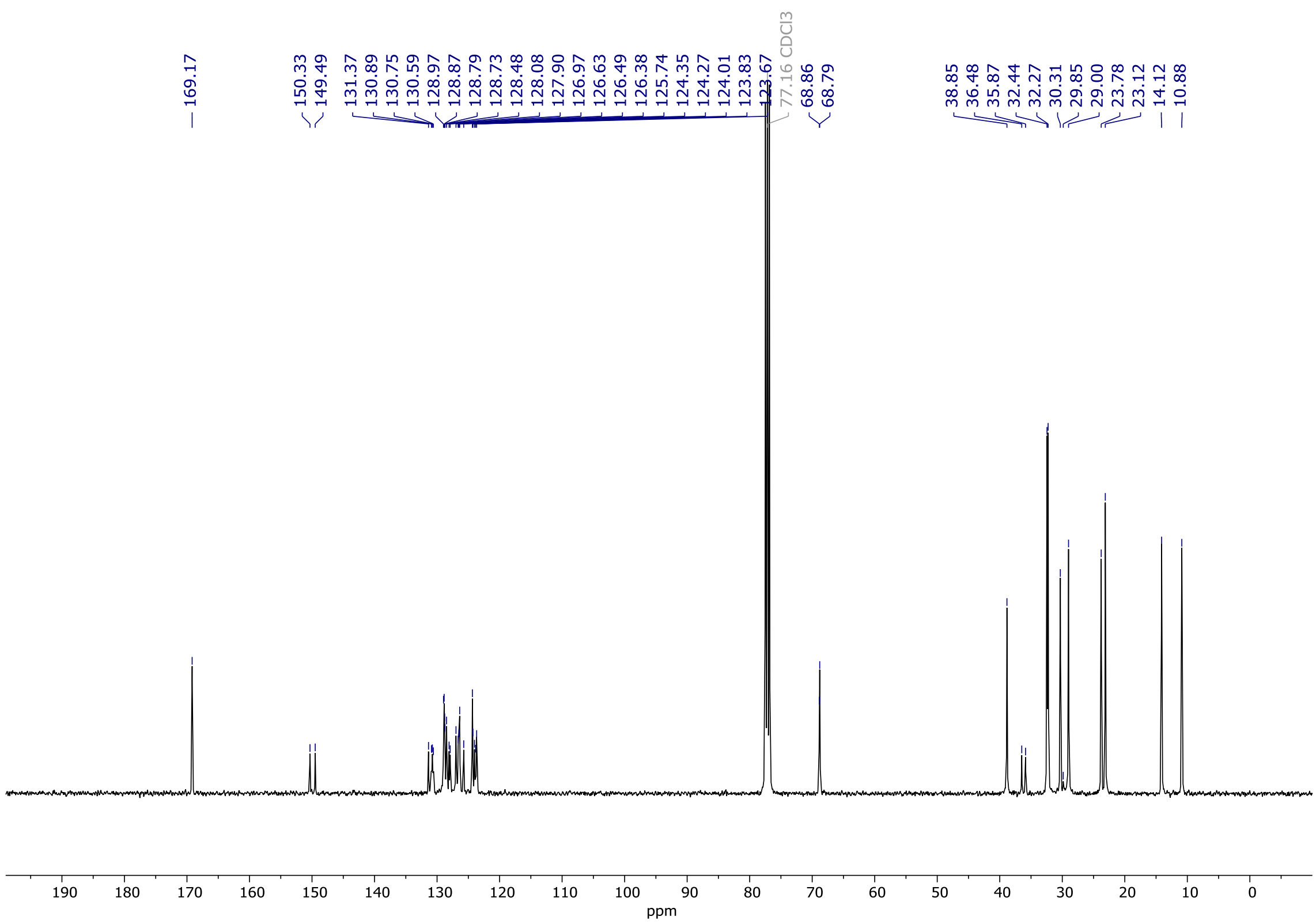

Figure S16. ^{13}C NMR (101 MHz, $CDCl_3$) of **NR-17**.

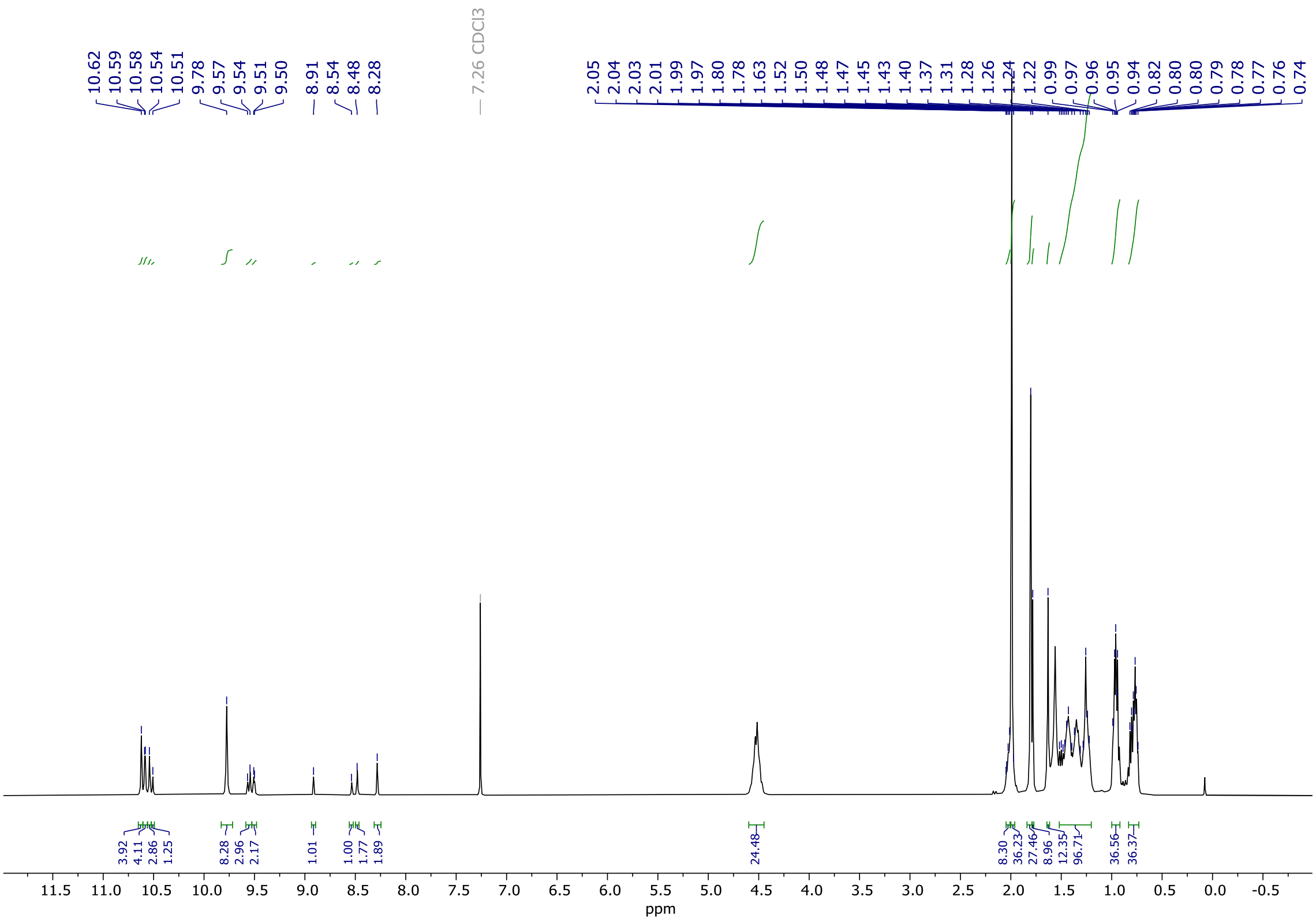

Figure S17. ^{1}H NMR (400 MHz, $CDCl_3$) of **NR-17-Bpin.**

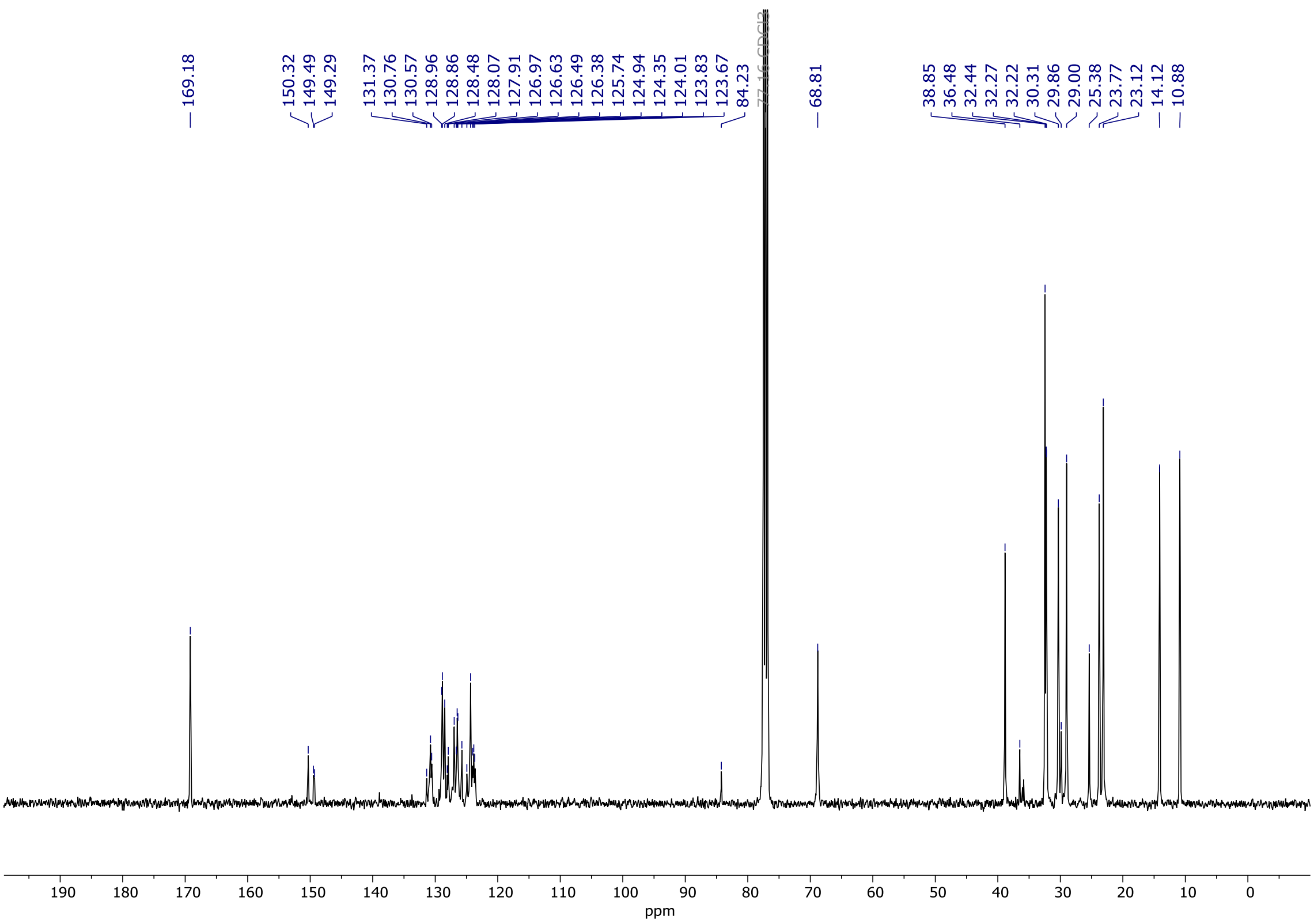

Figure S18. ^{13}C NMR (101 MHz, $CDCl_3$) of **NR-17-Bpin**.

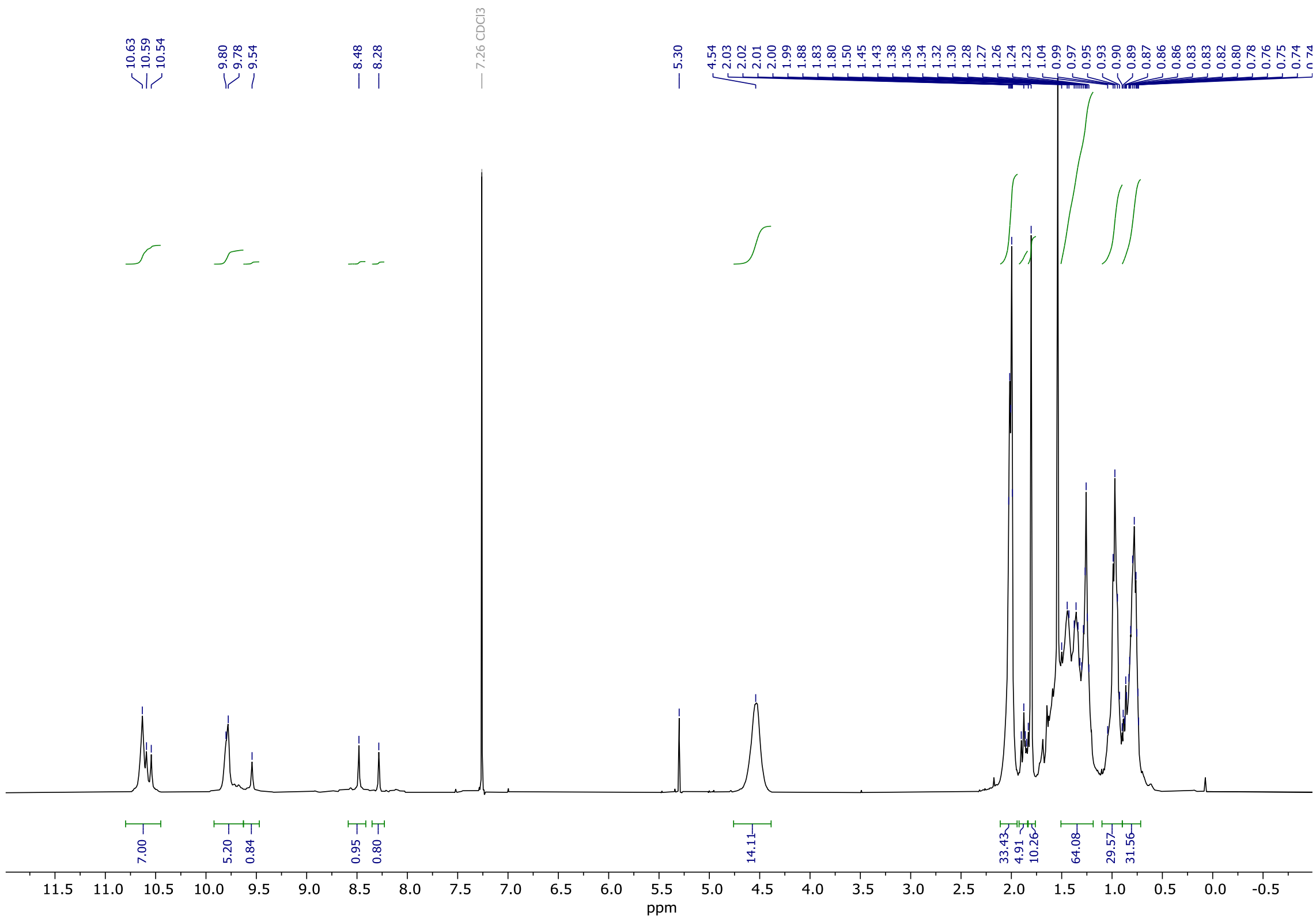

Figure S19. ^{1}H NMR (400 MHz, $CDCl_3$) of **NR-37**.

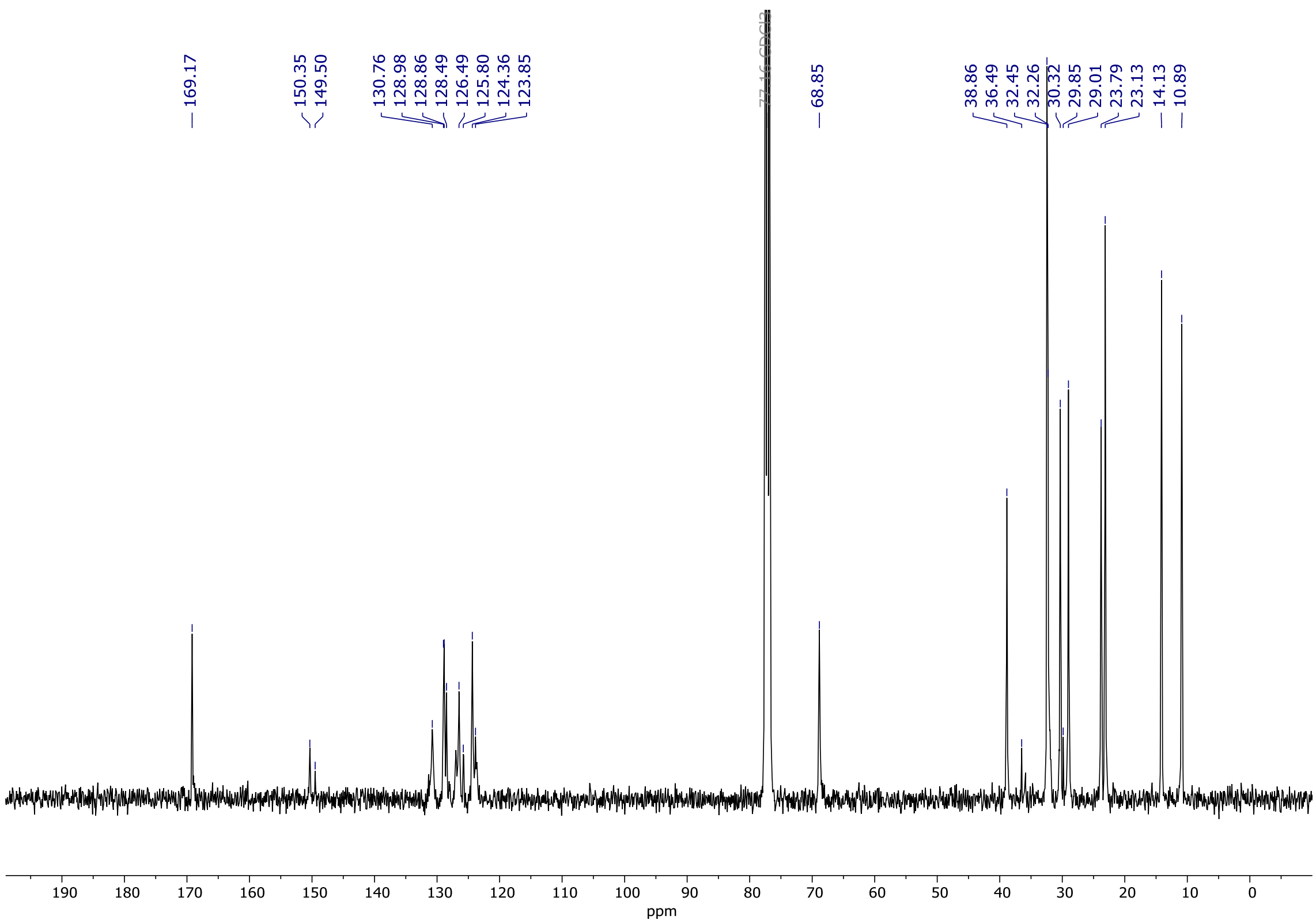

Figure S20. ^{13}C NMR (101 MHz, $CDCl_3$) of **NR-37**.

References

[1] R. K. Dubey, M. Melle-Franco, A. Mateo-Alonso, *J. Am. Chem. Soc.* **2021**, *143*, 6593-6600.

[2] R. K. Dubey, M. Marongiu, S. Fu, G. Wen, M. Bonn, H. I. Wang, M. Melle-Franco, A. Mateo-Alonso, *Chem* **2023**, *9*, 2983-2996.

[3] M. Fischer, J. Georges, *Chem. Phys. Lett.* **1996**, *260*, 115-118.
